# A halophilic *Chromohalobacter* species from estuarine coastal waters as a detoxifier of manganese, as well as a novel bio-catalyst for synthesis of n-butyl acetate

**DOI:** 10.3389/fmicb.2023.1159018

**Published:** 2023-04-12

**Authors:** Flory Pereira, Savita Kerkar, Dominic Savio Dias, Vivekanand V. Gobre

**Affiliations:** ^1^Department of Microbiology, P. E. S’s R. S. N. College of Arts and Science, Ponda, India; ^2^School of Biological Sciences and Biotechnology, Goa University, Taleigão, India; ^3^Department of Chemistry, P. E. S’s R. S. N. College of Arts and Science, Ponda, India; ^4^School of Chemical Sciences, Goa University, Taleigão, India

**Keywords:** *Chromohalobacter* sp., estuarine, manganese, detoxifier, bioremediation, bio-catalyst

## Abstract

Anthropogenic pollution due to ferro-manganese ore transport by barges through the Mandovi estuary in Goa, India is a major environmental concern. In this study a manganese (Mn) tolerant, moderately halophilic *Chromohalobacter* sp. belonging to the family Halomonadaceae was isolated from the sediments of a solar saltern adjacent to this Mandovi estuary. Using techniques of Atomic absorption spectroscopy, Scanning electron microscopy-Energy dispersive X-ray spectroscopy, Fourier-transform infrared spectroscopy and Atomic Force Microscopy, the *Chromohalobacter* sp. was explored for its ability to tolerate and immobilize Mn in amended and unamended media with 20% natural salt concentration (w/v). In aqueous media supplemented with 0.1 mM Mn, the *Chromohalobacter* sp. was capable of sequestering up to 76% Mn with an average immobilization rate of 8 mg Mn /g /day. Growth rate kinetic analysis using Gompertz mathematical functions was found to model the experimental data well. The model inferred that the maximum growth rate of *Chromohalobacter* sp. was at 10% natural salt concentration (w/v). The *Chromohalobacter* sp. was further found to be multimetal tolerant showing high tolerance to Iron (Fe), Nickel (Ni) and Cobalt (Co), (each at 4 mM), and tolerated Manganese (Mn) up to 6 mM. Morphologically, the *Chromohalobacter* sp. was a non-spore forming, Gram negative motile rod (0.726 μ× 1.33 μ). The adaptative mechanism of *Chromohalobacter* sp. to elevated Mn concentrations (1 mM) resulted in the reduction of its cell size to 0.339 μ× 0.997 μ and the synthesis of an extracellular slime, immobilizing Mn from the liquid phase forming Manganese oxide, as confirmed by Scanning Electron Microscopy. The expression of *Mnx* genes for manganese oxidation further substantiated the finding. This bacterial synthesized manganese oxide also displayed catalytic activity (∼50% conversion) for the esterification of butan-1-ol with CH_3_COOH to yield n-butyl acetate. This *Chromohalobacter* sp. being indigenous to marine salterns, has adapted to high concentrations of heavy metals and high salinities and can withstand this extremely stressed environment, and thus holds a tremendous potential as an environmentally friendly “green bioremediator” of Mn from euryhaline environments. The study also adds to the limited knowledge about metal-microbe interactions in extreme environments. Further, since *Chromohalobacter* sp. exhibits commendable catalytic activity for the synthesis of n-butyl acetate, it would have several potential industrial applications.

## Introduction

Naturally occurring hypersaline ecosystems of terrestrial as well as marine origin are an active, pivotal and vital part of planet earth. The interplay of biological, geological and chemical processes in this distinctive ecosystem not only impact the environment and society, but also have an effect on the economy (Shadrin and Anufriieva, 2013). Many hypersaline niches including naturally occurring saline water bodies, salt pans and salt marshes are frequently polluted due to the entry of elevated concentrations of metals from anthropogenic sources during the high tide. In solar salterns, when salt crystallizes the water evaporates and gets dense and metal concentrations increase. Unfortunately, heavy metals cannot be destroyed or degraded, unlike organic matter which can be easily converted to harmless end products. In such environments the native halophiles play a major role in mitigation of metal toxicity since they are able to survive under polyextremophilic conditions of salt stress, metal stress, alkalinophilic as well as thermophilic conditions (Ventosa et al., 1998). Some well-known examples of metal-tolerant bacteria are those belonging to the genera *Flavobacterium*, *Alcaligenes*, *Moraxella*, *Pseudomonas*, *Vibrio*, *Xanthomonas*, *Micrococcus*, *Aeromonas*, *Acinetobacter*, and *Spirochaeta* (DasSarma and DasSarma, 2012). Bacteria, fungi and algae from such environments can accumulate metal ions in their cells to concentrations much higher than the background concentrations of these metals (Coral et al., 2005). Zhang and Majidi (1994) evaluated microbes from extreme environments exposed to high salinity and metal stress and showed that the tolerance of microorganisms to heavy metals depended on their exposure to metals as well as their inherent physiological and/or genetic response mechanisms (Nagel and Voigt, 1989; Devars et al., 1998).

The Ribandar solar saltern from Goa, India, is one such classic example of a dual stressed environment subjected to high salt as well as high metal concentrations. It is located on the banks of the Mandovi estuary which receives an input of agricultural, industrial and domestic wastes, as well as an influx of spilled ferro-manganese ore during barge transport. Pollution studies of this estuary have been carried out by several researchers which indicate a continuous exposure of the saltern organisms to heavy metals (Kamat and Sankaranarayanan, 1975; Zingde et al., 1976; Alagarsamy, 2006; Krishnan et al., 2007). Kuppusamy and Hussain (2021) in their book chapter “Seasonal Variation for Trace Metals Contamination of Groundwater Using GIS Technology in Pissurlem, Sonshi, Cudnem, Velguem, Surla Watersheds, North Goa District, Goa State, India” reported findings of Mn in groundwater of villages in North Goa and observed that the Mn content in ground water increased during the summer season. Rodrigues et al. (2021) reported elevated levels of Mn in the Zuari estuary which is a part of the Mandovi Estuary in the State of Goa. Values as high as 4058 ppm at the surface and 3488 ppm at the bottom were reported. On similar lines, metal levels in sediments were 4.72%, 850 ppm, 95 ppm, and 45 ppm for Fe, Mn, Zn, and Cu, respectively, which exceeded average shale values, indicating anthropogenic pollution. Further, bioaccumulation of Fe, Mn, Zn, and Cu was reported in the edible bivalve *Saccostrea cucullata*, impacting their growth and reproductive ability. The prevalence of metals, such as Fe, Mn, and Zn, in hard shells of bivalves above the permissible limits was also attributed to anthropogenic pollution in the Zuari Estuary, due to iron ore mining. This has resulted in the emergence of bacterial strains that have acclimatized and developed tolerance to high concentrations of metals (Pereira et al., 2012). In our earlier studies we showed that this Mandovi estuary falls in the moderately contaminated category according to the geoaccumulation index calculation (Pereira et al., 2013). The increase in Fe concentration in this estuary was reported to be 8–17 times higher in the salt making season, whereas the increase in Mn was 34–54 times more, with a resultant amplification in the number of metal tolerant bacteria in the adjoining saltern. Based on the physiological importance of metals they may be categorized as: (1) non-toxic, e.g., Ca and Mg; (2) toxic, e.g., Hg and Cd; and (3) essential in small amounts but harmful above a certain level, e.g., Mn. The concentration of Mn in the Mandovi estuary falls in the harmful category. Mitigation of such toxic levels can be brought about by manganese tolerant bacteria. Swanson et al. (2023) observed that anoxic conditions resulting from overgrowth of algae impacted metal mobility by influencing the oxidation states and bonding capability of Fe and Mn at the interface of water and sediment at depths above 2.5 cm. In our study, the Ribandar Salt pan is inundated with microbes and algae during the non-salt-making season, creating an anoxic environment which would be conducive to alteration of the oxidation states of Fe and Mn, thereby causing temporary release of Fe and Mn into the surrounding which could influence metal-microbe bond formation and complexation.

Palmer et al. (1986) and Bargar et al. (2005) reported strains belonging to the genera *Brevibacillus*, *Arthrobacter*, and *Sphingomonas* capable of manganese oxidation. Manganese tolerant bacteria such as *Bacillus* species capable of Manganese oxidation have been isolated from various environments including coastal areas (Francis and Tebo, 2002), deep-sea vents (Dick et al., 2006), and the oxygen limited zone of the Black Sea (Stewart et al., 2007). Manganese oxidation by *Halomonas* sp. has been reported in the Carlsberg ridge (Fernandes et al., 2005). The marine *Pseudomonas* sp. strain S-36 was reported to use manganese oxidation as a means for obtaining energy for CO_2_ fixation (Kepkay and Nealson, 1987).

Hence, we hypothesize that indigenous manganese tolerant bacteria could play an important role in mitigation of manganese toxicity. Further, these microorganisms could be harnessed for biosynthesis of metal oxides, since green chemistry is increasingly being sought as a low-cost, eco-friendly, functionally effective option. Metal oxides such as manganese oxide have many applications, in being used as bio-catalysts in the chemical industry (Kratošová et al., 2019). Synthesis of n-butyl acetate is normally carried out using metal oxides or mineral acids as catalysts (Nagvenkar et al., 2015). The n-butyl acetate, a colorless ester with a fruity odor finds applications in the food industry as a fruity flavoring agent in the manufacture of ice creams, baked goods and confectionery. It is used in agriculture as a natural trap for insects because it has odor mimicking alarm pheromones. It is a highly versatile solvent, capable of reducing viscosity and improving solvency and so it has many pronounced industrial and commercial applications, viz as an extraction solvent for lacquers and paints, in pharmaceutical and chemical industries. It finds uses as an ingredient in cosmetics, perfumes, cleaning products and car care products. It is also used in the leather industry, and for manufacture of adhesives and hardened coatings. PBLFs (Petroleum-based liquid fuels) require blending with biofuels in order to stem the emissions of particulates and minimize the utilization of PBLFs. In this regard, n-butyl acetate is considered to be the best candidate as reported in the literature (Wang et al., 2023). The n-butyl acetate is prepared by refluxing acetic acid and butan-1-ol, utilizing inorganic liquid or solid acid catalysts such as sulfuric acid or *p*-toluene sulphonic acid (Gangadwala et al., 2003; El-Sharkawy and Al-Shihry, 2004; Blagov et al., 2006; Martins et al., 2011). However, synthesis of n-butyl acetate by chemical methods though economical is not environment friendly, resulting in other problems such as environmental pollution and corrosion (Jermy and Pandurangan, 2006). Biological synthesis methods by the use of microorganisms offers a permanent solution to environmental pollution due to their safety and eco-friendly characteristics.

In the present study we have isolated and characterized manganese tolerant bacteria capable of manganese oxidation, and evaluated their potential in Mn removal, as well as utilized the manganese oxide formed as a biocatalyst. This is the first study to show that a moderately halophilic *Chromohalobacter* sp. from a marine saltern is capable of Mn oxidation and bioremoval of Mn. The study gains further relevance due to the fact that oxides of manganese (MnO_*x*_) are extremely reactive (Tebo et al., 2004) and may therefore adsorb other metals, thereby regulating the high concentration of other metals in the salterns by chemisorption, thus augmenting the overall bioremoval of the other metals from the environment. Additionally in our present study, taking into account the varied applications of n–butyl acetate, we have synthesized and characterized n–butyl acetate using a biocatalytic route employing *Chromohalobacter* sp. as a catalyst.

## Materials and methods

### Media and reagents

All the acids used were procured from Merck. All solvents and reagents were of ACS grade of 99% purity. De-ionized water was obtained from Milli-Q water purification system. Nutrient broth and agar were procured from Hi Media Laboratories Pvt. Ltd., Bombay, India. Analytical grade MnCl_2_ was procured from Merck. All metal solutions were filter sterilized through a 0.2 μ membrane filter.

### Physico-chemical analysis of saltern sediment and water

**FIGURE 1:**
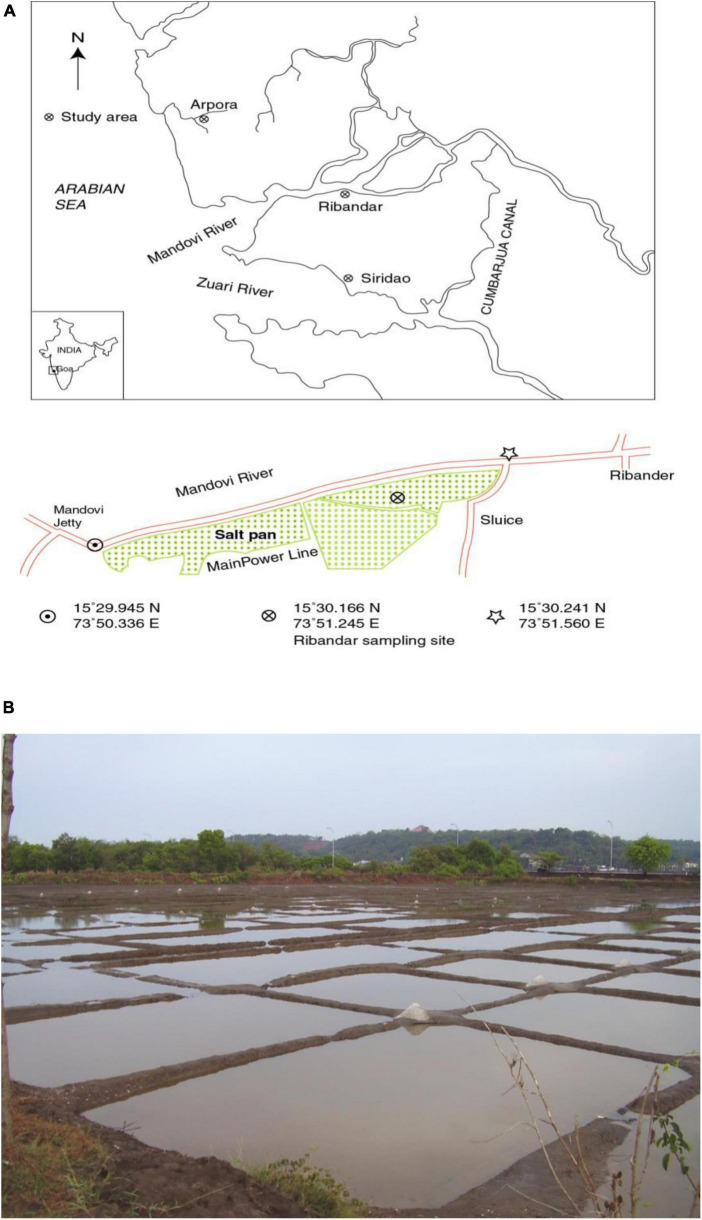
**(A)** Map showing location of Ribandar saltern in Goa, India and **(B)** study site.

Soil samples were collected from a crystallizer pond (15° 30.166 N and 73° 51.245 E) of the Ribandar solar saltern situated along the Mandovi estuary in Goa, India (Figures 1A, B). Temperature and salinity measurements of the sediments were carried out on site at low tide before sunrise. Water temperature was measured to a precision of 0.1°C using a field thermometer (76 mm immersion, ZEAL, England). Salinity was measured using a hand held refractometer (S/Mill-E, ATAGO Co. Ltd., Japan) calibrated to zero with distilled water. Whenever salinity was above 100 psu, the sample was diluted with distilled water (1:5) and subsequently analyzed. The pH and Oxidation/reduction potential (Eh) were measured in the laboratory by Thermo Orion model 420A, (USA) as described in Orion instruction manual. The Mn concentration in the overlying water and sediment was also measured. The procedure followed was, the solvent extraction method using Ammonium Pyrrolidine Di-thio Carbamate and Methyl Isobutyl Ketone (APDC–MIBK) (Brooks et al., 1967). In brief, 250 ml of water sample was acidified to pH 2 using 2N HNO_3_ and then the pH was adjusted to 4.5 using liquid ammonia. The sample was fractionated into aqueous and organic layers with 1.25 mL of APDC and 2.5 mL MIBK in a separating funnel. The extraction was carried out thrice and the organic fraction was pooled, acidified with 2.5 mL of 4 N HNO_3_, and used for the analysis. For the sediment samples, the method described by Balaram et al. (1995) was followed. A total of 0.2 g dried powdered sediment sample was digested in 10 mL of concentrated HF, HNO_3_, and HClO_4_ (7:3:1) and mixed in a sealed Teflon digestion bomb. The residue was dried on a hot plate in a fume hood, acidified with 5 mL of the acid mix, 2 mL of concentrated HCl and 10 mL of HNO_3_. Finally the volume was adjusted to 50 ml with double distilled water. All the acids used were of 99.9% purity and procured from Merck. The concentration of Mn was analyzed with an atomic absorption spectrophotometer (AAS) GBC 932AA model, (Australia). The blank corrections were applied wherever necessary and the accuracy was tested using standard reference material MAG-1 (United Geological Survey, Revised March 1995) (Abbey and Dickson, 1983; Gladney and Roelandts, 1987; Govindaraju, 1994).

### Isolation of Mn tolerant bacteria

The Mn tolerant culture (labeled FSK3) was isolated from the Ribandar saltern sediment by serial dilution and plate count method. Serial dilutions were prepared using 1 g of saltern sediment (0–5 cm depth) and plated out on (25%) i.e., quarter strength Nutrient Agar plates (100% corresponds to 13 g nutrient broth in 1 L^–1^ with 2% agar-agar as the solidifying agent) amended with 2 mM Mn and 20% crude salt (w/v). All stock solutions of MnCl_2_ used were filter sterilized through a 0.2 μ cellulose acetate (Millipore) membrane. The isolate was further screened for Mn tolerance from 1 to 5 mM. The purity of the culture was assessed by plating on solid media and observing colony morphology of isolated colonies, and further confirmed by Gram staining and observing under a microscope.

### Metal tolerance studies and growth kinetics

Toxicity studies of selected isolate (FSK3) to Manganese at concentrations of 0.01, 0.1, 1, 5, and 10 mM, respectively, and salinities ranging from 0 to 20% were carried out in 96 well sterile polystyrene microplates (non-treated, round bottom with lid). For the test, 200 μl of sterile 25% nutrient broth amended with Mn to a final concentration of 0.01, 0.1, 1, 5, and 10 mM, respectively, was inoculated with 20 μl of exponentially growing cultures (24 h old, optical density of ∼1.2 at 600 nm). Controls without Mn were maintained. All the experiments were conducted in triplicate. Lids of microplates were fixed with condensation rings, sealed tightly with laboratory film (Parafilm^®^ M) and incubated at 28°C. Optical density (OD) at 600 nm was noted using the Microplate Reader (Synergy 2 Bio-Tek) till the stationary phase was attained. OD was correlated to cell counts, by counting the number of cells under 400-× magnification with (BX51 Olympus microscope). Growth of the isolate at varying salinities of 0, 2, 10, and 20% and metal amendments from 0 to 10 mM Mn was recorded and growth kinetics calculated as per the growth rate models described in Zwietering et al. (1990).

### Identification and characterization of the Mn tolerant isolate

Selected Mn tolerant bacterial isolate (FSK3) was characterized based on its morphology, biochemical tests, and 16 S rRNA gene sequencing. Universal bacterial forward primer 5′CGAATTCGTCGACAACAGAGTTTGATCCTGGCTCAG-3′ and bacterial reverse primer 5′-CCGGGATCCAAGCTTA CGGCTACCTTGTTACGACTT-3′was used for amplifying 16 S rRNA gene. Purification of the amplified product was carried out using QIA quick PCR Purification Kit (QIAGEN, India) as recommended by the manufacturer. CFX 96, Real time system was used for detection and the product was analyzed using a sequencer. A segment of approximately 1500 bases of 16 S rRNA gene was amplified and sequenced. The sequence was compared with the sequence of strains belonging to the same phylogenetic group from the GenBank database using BLAST search of the National Centre for Biotechnology Information (NCBI).

### Evaluation of biosorption potential

To quantify the biosorption potential of Mn by isolate FSK3, 10 ml aliquots of 25% Nutrient broth having 20% salinity (w/v) and supplemented with Mn at 0.01, 0.1, 1, and 5 mM, respectively, were taken in 15 mL screw capped test tubes and inoculated with the test isolate FSK3 at an initial cell count of 2–4 × 10^7^ cells mL^–1^. To correct for chemical oxidation and metal losses due to autooxidation and adsorption on glass tubes, controls with media and metal but without the test organism were maintained. The tubes were incubated at 28 ± 2°C in the dark. Sample (1 mL) was removed immediately and again after 10 days of incubation for determining the total count, wet weight of biomass and remnant metal concentration in the liquid phase. Metal concentration was determined by atomic absorption spectrophotometry (AAS; GBC 932AA model) equipped with deuterium background corrections. Blank corrections were done. All experiments were carried out in triplicates and the acids used for analysis were procured from Merck.

### Alcian–blue staining for extracellular polymeric substances (EPS) production

Smears of FSK3 cells grown with 1 mM Mn metal amendments were prepared on a slide. Control without Mn amendments were also prepared. Further they were air dried and hydrated with distilled water. The slides were stained with 10 μL of 0.1 % alcian blue dye in acetic acid at pH 2.5 for 5 min., washed with water; dried and observed under oil immersion microscope (Decho, 2018).

### Light microscopy and SEM-EDS analysis

Bright field microscopy (BX51 Olympus microscope) at 1000-× magnification was used for examining morphological changes in the Gram stained smear. Scanning electron microscope (SEM) coupled with energy dispersive X-ray spectrometer (SEM-EDX) (JEOL JSM-5800 LV) was used to elucidate the chemical nature of the metal immobilized by the isolate and compare it with the control. For the test, bacterial isolate FSK3 was grown in 25% Nutrient broth amended with 1 mM Mn for 10 days. Cells grown without Mn served as a control. A smear of the cells was prepared on a coverslip and sequentially dehydrated in 20, 50, 75, and 90% acetone for 15 min each and finally dehydrated in 100% acetone for 30 min. The samples were processed for CPD (Critical point drying), sputter coated on a spi sputter coater with gold for 30 s and examined under a JEOL JSM-5800 LV SEM.

### Atomic force microscopy

For Atomic Force Microscopy (AFM) imaging, isolate FSK3 was grown on 0.25% nutrient agar plates amended with Mn at a concentration of 2 and 20 mM at 10% salinity. Untreated control without metal was also maintained. A single colony from each of the plates was resuspended in sterile saline, and 20 μL of the cell suspension was spread on a 1.0 cm × 1.0 cm mica sheet. Cells were air-dried and used for AFM analysis.

The experiment was carried out with AFM with trade name NTEGRA Prima mounted on an Olympus inverted optical microscope and operated with the Nano Technology Molecular Device Technology (NTMDT) controller, placed on a minus *k*-technology vibration isolator. This technique detects the van der Waals forces existing between a silicon probe and materials on surfaces (or the surface sample or biomaterial), scanned along the cantilever tip with a piezo scanner (maximum XYZ scan range of 10 μm × 10 μm × 3 μm). The cantilever tip was attached at one end to a cantilever which is built in at the other end. This technique can generate information detected on surface topography and mechanical properties at the nanometer scale. All data presented in this article was generated with NSG01 series cantilevers (single crystal silicon, N-type, resistivity 0.01–0.025 Ω-cm, antimony-doped), whose spring constant was about 40 N/m, with a maximum resonant frequency of 150 kHz and at a scan rate of 0.2–1 Hz. We performed experiments at 18°C in a dehumidified acoustic room. We employed non-contact atomic force microscopy (NC-AFM) modes; amplitude modulation, which gave height profile. We processed the data obtained in AFM measurements using open source software Gwyddion (Nečas and Klapetek, 2012; Klapetek et al., 2017). The height profile data was processed by subtracting plain surface data to eliminate tilt in the images and facet leveling.

### IR spectroscopy

Infra-red spectroscopy (IR) studies were performed using IR Prestige 21 Shimadzu in the region 400–4000 cm^–1^ using KBr as reference. For FTIR, the microbial cells of FSK3 grown without Mn (0 mM) and with amendments of 2 mM and 20 mM of Mn were ground with KBr (FTIR grade) and pressed to a pellet. The pellet was examined against a blank KBr pellet.

### Screening for manganese tolerance genes

DNA was isolated using bacterial genomic DNA prep kit (Chromous Biotech RKT11/12) from a single colony of isolate FSK3 grown with and without 1 mM Mn amendments, respectively, on 25% Nutrient agar plates with 20% crude salt (w/v). Bacterial (manganese tolerance) *Mnx*G genes were screened and amplified by using degenerate primers *Mnx*GUf (CAGRTGRATRTGCTGGCCGAT) and *Mnx*GBr1 (RAAIARRTGTRCRTGRAARAA) (Dick et al., 2006). The PCR protocol was as follows: 30 cycles of 94°C for 30 s, 45°C for 30 s, and 60°C for 1 min, followed by 1 cycle of 72°C for 15 min. The amplified product was quantified using a Nanodrop spectrophotometer and the samples were equilibrated to get a final concentration of 25 ng/μL. A total 10 μL of the sample was loaded in each well and subjected to electrophoresis on a 0.8 % agarose gel using TBE buffer at 100 V for 2 h. Broad range DNA markers (BIORAD) were used. DNA bands were visualized and photographed using Alliance 4.7 Gel doc system.

### Bio-catalytic synthesis of n–Butyl acetate

CH_3_COOH (15 mL) was heated to 70°C in a 50 mL round bottomed flask. Later, 10 mL of preheated butan-1-ol was added to the reactor. To it 50 mg of isolate FSK3 was added. Contents were stirred continuously under reflux at 80°C. Aliquots of the reaction mixture were analyzed volumetrically to estimate the % conversion (Peter et al., 2012; Nagvenkar et al., 2015).


%Conversion(aceticacid)=[C-iC/tC]ix100

where, C_*i*_ signifies the initial concentration of the acid and C_*t*_ is concentration of acid at time t.

## Results

### Physico-chemical properties of saltern water and sediment

Physico- chemical parameters (temperature, pH, conductivity, and salinity) of soil samples collected during the salt-making season from the Ribander solar saltern are as shown in Table 1. Mean, Analysis of Variance (ANOVA), and Correlation coefficient were done using the statistical package of Microsoft Office Excel 2007. The temperature in the overlying water varied between 22.2 ± 1.6 and 35 ± 1.2°C (Mean = 26.6 ± 3.6°C) and in the sediment it ranged between a minimum of 24 ± 1°C (at 0–5 cm depth) and a maximum of 35.7 ± 1.4°C (at 5–10 cm depth) (Mean = 29.6 ± 3.8°C). The pH of the overlying water was slightly alkaline varying between 7.2 and 8.0, however, the pH in the sediment pore water was generally acidic and ranged between 6.4 and 6.5 at a depth of 0–5 cm and 5.7 to 6.6 at a depth of 5–10 cm, respectively. Salinity of the water ranged from 4.6 ± 8 to 28.5 ± 9%. The highest salinity (29.6 ± 9%) encountered in the water was in the month of April when salt-making was at its peak. During salt-making season the salinity values in the sediment averaged about 10.8 ± 4% at 0–5 cm, and 7.3 ± 4% at 5–10 cm. The redox potential in the sediments followed the general paradigm of decreasing with depth. Manganese values ranging between 1.56 ± 0.2 mgL^–1^ and 1.64 ± 0.2 mgL^–1^ were recorded in the overlying water. Manganese concentrations in the saltern sediment ranged from 0.59 ± 0.2% to 1.06 ± 0.03 at 0–5 cm depth and 0.69 ± 0.3 to 0.99 ± 0.2% at 5–10 cm depth.

**TABLE 1:** Physico-chemical parameters during the salt-making season (Mean ± S.D).

Parameters	Saltern sample	Minimum	Maximum
Salinity (%)	water	4.6 @ 8	28.5 @ 9
	0–5 cm sediment	3.2 @ 2	14.8 @ 1.3
	5–10 cm sediment	2.3 @ 0.6	13.2 @ 2.5
Temperature (°C)	Water	22.2 @ 1.6	35 @ 1.2
	0–5 cm sediment	24 @ 1	35 @ 1.7
	5–10 cm sediment	24.2 @ 0.9	35.7 @ 1.4
pH	Water	7.2 @ 0.5	8.0 @ 0.1
	0–5 cm sediment	6.4 @ 0.1	6.5 @ 0.1
	5–10 cm sediment	5.7 @ 0.1	6.6 @ 0.1
Eh (mV)	Water	43.2 @ 4.3	122 @ 17.8
	0–5 cm sediment	-10.3 @ 10.2	27.4 @ 16
	5–10 cm sediment	-10.8 @ 2.7	27.2 @ 10.6
Mn (mgL^–1^)	Water	1.56 @ 0.2	1.64 @ 0.2
Mn (%)	0–5 cm sediment	0.59 @ 0.2	1.06 @ 0.03
	5–10 cm sediment	0.69 @ 0.3	0.99 @ 0.2

### Isolation of Mn tolerant bacteria

The total counts of Mn-tolerant bacteria varied between 10^5^ and 10^6^ cfu g^–1^ sediment. From the 127 isolates which were screened for metal tolerance, a total of 87% showed tolerance to Mn at concentrations as high as 4 mM at salinities ranging from 2 to 20%. Isolate FSK3 was selected because it could grow well at a pH ranging between 6.0 and 8.5 and a salinity ranging from 0.35 to 20% (w/v) with manganese amendments as high as 20 mM. Optimum growth was at 28°C, pH 7.4, and 10% crude salt (w/v). The organism was motile, rod shaped and Gram negative. It produced a light orange pigment which increased with an increase in Mn concentration. The size of the colonies on solid media increased with Mn amendments up to 2 mM and then decreased at higher metal concentration (Supplementary Figure 1). When grown in media amended with 1 mM Mn, the isolate precipitated Mn in the form of Manganese oxide, which was seen as a dark brown precipitate in broth culture. The isolate also displayed multi-metal tolerance to Pb (up to 6 mM), Fe (3 mM), Co (3 mM), Ni (3 mM), Cd (0.5 mM), Zn (0.4 mM), and Hg (0.1 mM), respectively.

### Metal tolerance studies and growth kinetics

**FIGURE 2:**
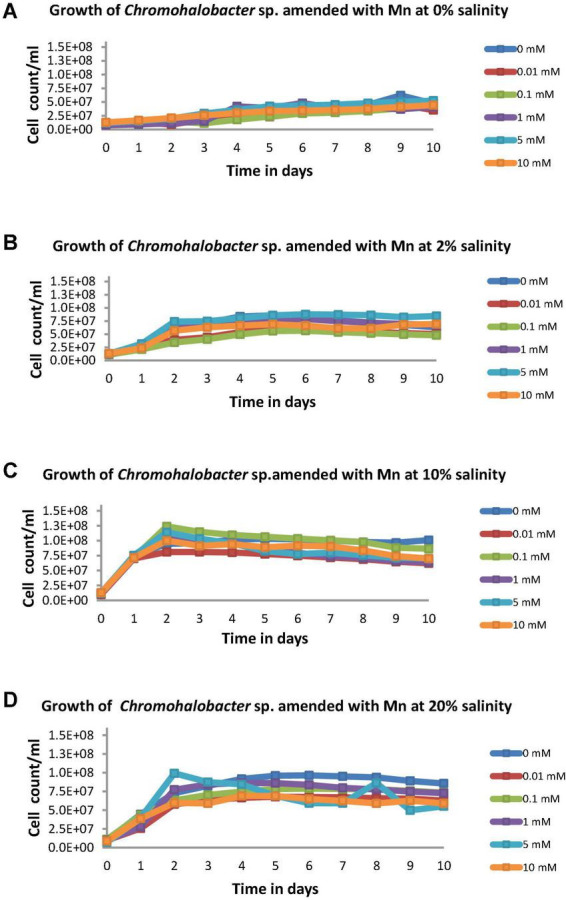
Cell counts of *Chromohalobacter* sp. at **(A)** 0% salinity, **(B)** 2% salinity, **(C)** 10% salinity, and **(D)** 20% salinity each with metal concentrations varying from 0 to 10 mM.

Growth of isolate FSK3 at varying concentrations of metal between 0.01 and 5 mM and salinities of 0 to 20% (Figure 2) indicated that isolate FSK3 failed to grow both at low Mn (0.01 mM) as well as high Mn (5 mM) concentrations in the absence of salt and showed a prolonged lag phase (Figure 2A). At 2% salinity, the isolate was able to grow and reach cell counts of about 7 x 10^7^ cfu mL^–1^, after an initial lag phase of about 24 h (Figure 2B). An increase in crude salt to 10% (w/v) had an immediate growth enhancing effect at all concentrations of Mn tested between 0.01 mM and 5 mM (Figure 2C). However, at 10% salinity, growth was best with 1 mM Mn and the cell counts reached 1.3 x 10^8^ cfu mL^–1^. High crude salt concentration of 20% (w/v) resulted in a reduction in the cell counts at even low concentrations of Mn such as 0.01 mM and 0.1 mM (Figure 2D). Surprisingly, further increase in metal concentration to 1 mM resulted in an increase in counts from 0.8 x 10^7^ to 1.1 x 10^7^ cfu mL^–1^. When metal concentration was increased to 5 mM, growth increased from 1.2 x 10^7^ to 2 x 10^7^ cfu mL^–1^.

Optical data vs. time in days was modeled using mathematical function (Supplementary Figure 2). The Gompertz model fitted the observed data better than the Logistic model. With increasing salinity, the growth kinetics of bacteria showed an increase in growth rate with simultaneous enhancement of growth rate constant irrespective of concentration of metal (Table 2).

**TABLE 2:** Parameters (A, λ, and μm) of non-linear fit for various functions used in the article.

Salinity (%)	A*^o^*	μ_*max*_	λ
0	0.32	0.023	–2.27 (Logistic)
	0.39	0.024	–2.27 (Gompertz)
2	0.32	0.0913	0.088
	0.32	0.0933	–0.013
10	0.40	0.29	0.711
	0.40	0.36	0.75
20	0.36	0.13	0.30
	0.36	0.14	0.30

Logistic and Gompertz modeling of growth kinetics of Chromohalobacter sp. at varying salinities with manganese amendments.

### Identification and characterization of the Mn tolerant isolate

Preliminary identification was done using biochemical media. In biochemical media, isolate FSK3 could only utilize fructose, dextrose, melibiose, sucrose, L-arabinose, esculin, citrate, malonate, and sorbose. Initially it was tentatively identified as *Pseudomonas* sp.

16 S rRNA sequencing revealed the identity of FSK3 to be *Chromohalobacter* sp. Distance Matrix of isolate FSK3 constructed with ∼1500 bp of 16 S rRNA sequences based on Nucleotide Sequence Homology (Using Kimura-2 Parameter and NCBI GenBank and RDP database) revealed 97% similarity with *Chromohalobacter salexigens* and 94% to *Chromohalobacter beijerinckii* (Table 3). The 16 S rRNA sequence was deposited in GenBank with Accession Number: JQ312118.

**TABLE 3:** Alignment view using combination of NCBI GenBank and RDP database.

ID	Alignment results	Sequence description
FSK3	0.97	Isolate *FSK3* 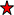
EU122306	0.95	*Pseudomonas beijerinckii strain ATCC 19372T*
AJ295146	0.95	*Chromohalobacter salexigens strain TPSV 101*
AY505516	0.95	*Chromohalobacter sp. 2-NaCl*
AB021386	0.95	*Chromohalobacter marismortui*
NR-025431	0.95	*Chromohalobacter sp. MAN K24*
AB166934	0.95	*Chromohalobacter marismortui strain GSP58*
DQ789389	0.95	*Chromohalobacter israelensis strain Ba1*
U78719	0.95	*Chromohalobacter salexigens strain NJ2*
EU221419	0.95	*Chromohalobacter israelensis*
AF211862	0.93	*Halomonas elongata strain DSM 3043*

### Manganese bioremoval potential of *Chromohalobacter* sp.

**FIGURE 3:**
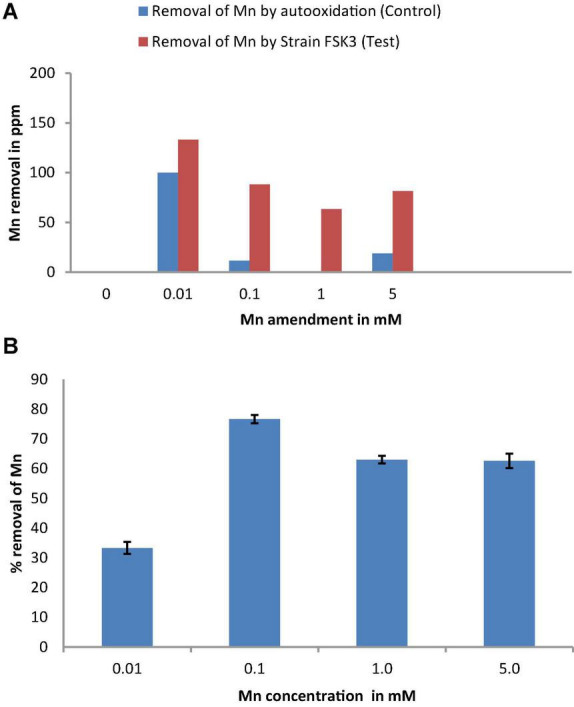
**(A)** Mn removal by isolate FSK3 (*Chromohalobacter* sp.) at 0.01, 0.1, 1, and 5 mM Mn concentrations. **(B)** Percentage removal of Mn by isolate FSK3 (*Chromohalobacter* sp.) at 0.01, 0.1, 1, and 5 mM Mn concentrations.

Percentage of Mn bioremoval was calculated relative to auto oxidation occurring in controls (Figure 3). Even though the bioremoval of Mn by *Chromohalobacter* sp. appeared to be high at a concentration of 0.01 mM Mn, a large fraction was due to auto-oxidation (Figure 3A). Optimum Mn bioremoval occurred at 0.1 mM Mn concentration with an efficiency of 76% (Figure 3B). With an increase in Mn concentration to 1 mM there was a slight decline in Mn bioremoval to 63%, which further declined to 62% at 5 mM Mn.

### Alcian-blue staining for extracellular polymeric substances (EPS) production

**FIGURE 4:**
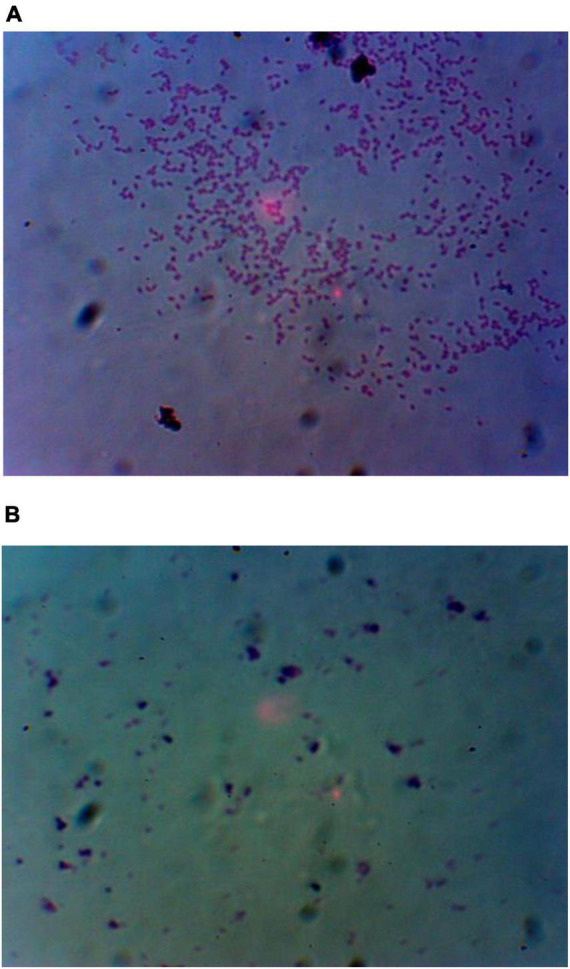
**(A)** Alcian blue staining for detection of EPS in FSK3 (*Chromohalobacter* sp.) without Mn treatment and **(B)** with 1 mM Mn treatment.

Staining with Alcian blue was done to study if EPS was produced as a mechanism for metal tolerance. Cells of *Chromohalobacter* sp. treated with Mn showed a slimy EPS layer which was detected by Alcian blue staining (Figure 4). The control cells remained pink (Figure 4A) while the Mn treated cells stained blue due to EPS production (Figure 4B).

### Light microscopy and SEM-EDS analysis

**FIGURE 5:**
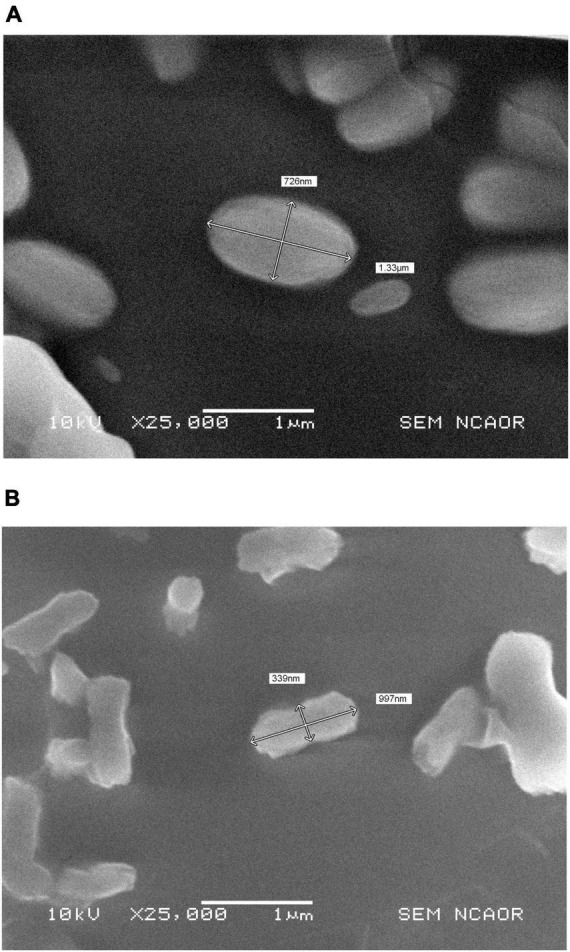
**(A)** SEM-photomicrographs showing *Chromohalobacter* sp. without Mn treatment and **(B)** reduction in cell size of *Chromohalobacter* sp. treated with 1 mM Mn.

Scanning electron microscope images of Mn treated *Chromohalobacter* sp. and untreated control are shown in Figures 5A, B. Morphologically, the Mn unamended cells (control) appeared normal, having a cell size of 0.726 μm x 1.33 μm. Exposure to 1 mM Mn concentrations resulted in a reduction in cell size to 0.339 μm x 0.997 μm, inclusive of the slime layer around the cell.

**FIGURE 6:**
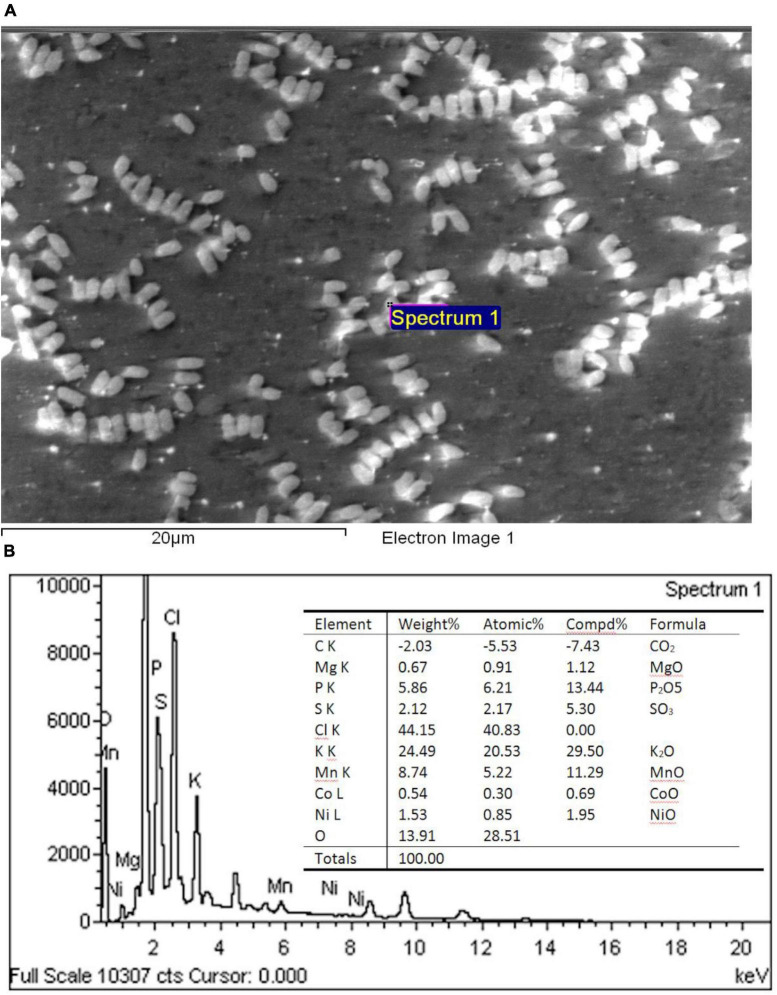
**(A)** SEM–photomicrograph of *Chromohalobacter* sp. when grown with 1 mM Mn and **(B)** energy dispersive X-ray spectrometric analysis of the manganese oxide produced by *Chromohalobacter* sp. when grown with 1 mM Mn.

Electron micrographs confirmed the reduction in the cell size of Mn treated cells. The cells also appeared rough and distorted, but cell lysis was not visible. EPS was visible in the SEM photograph (Figure 6A). Manganese oxide was not detected by EDS as deposits on individual bacterial cells. However, SEM-EDS analysis of the brown precipitate obtained in the culture flask confirmed the precipitate to be manganese oxide deposits within the bacterial EPS secretions (11.65% MnO) (Figure 6B).

### AFM analysis

**FIGURE 7:**
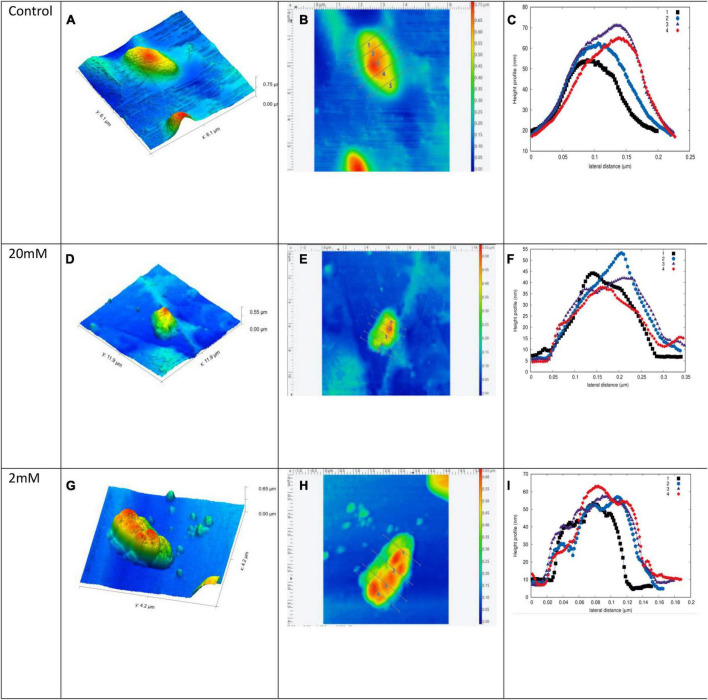
*Chromohalobacter* sp. single cell surface topography as mapped under AFM. **(A–C)** Control without any treatment. *Chromohalobacter* sp. cells treated with 20 mM concentration of Mn **(D–F)**. *Chromohalobacter* sp. cells treated with 2 mM concentration of Mn **(G–I)**, where **(A,D,G)** indicate NC-AFM 3D height profile, **(B,E,H)** linear path mapping across single cell, **(C,F,I)** height profile graph of surface roughness for *Chromohalobacter* samples. Supplementary Figure 3 includes high resolution AFM images of single cell surface topography of *Chromohalobacter* sp. without Mn and with Mn treatment.

Single cell surface topography of *Chromohalobacter* sp. was mapped under AFM. Test sample comprised of *Chromohalobacter* sp. grown on media amended with 2 and 20 mM Mn, respectively. The control was *Chromohalobacter* sp. without any metal treatment. NC-AFM 3D height profile, linear path mapping across single cell was done. The three-dimensional imaging of the *Chromohalobacter* sp. treated with 2 and 20 mM Mn clearly shows morphological/topological changes. The plotted graph for cell height and surface roughness indicated a high degree of roughness on manganese treated cells compared to control cells (Figure 7 and Supplementary Figure 3). The AFM measurements were carried out under ambient conditions (air) at a non-/contact intermittent mode to get advantage of the harmonic oscillator behavior of the AFM probe, The height information was obtained from a linear map across a sample cell in the control cell. The data predicts that the control cell had a length of ∼3 μm, width of 2 μm and a height of about 50 nm (Figures 7A–C). With 2 mM Mn amendment the cell length was 2.5 μm, width was 1.6 μm and height was 50 nm (Figures 7G–I), whereas for 20 mM the cell length was 4 μm, width was 3 μm and height was 40 nm (Figures 7D–F). The linear path across the control cell showed smooth topological distribution, with vertical height distribution (ΔZ) of 15 nm. However, the Manganese–treated samples showed ΔZ distribution of ∼55 and ∼45 nm, respectively, with 2 mM and 20 mM Mn amendments. The data indicated higher morphological changes with 2 mM Manganese treatment.

### IR spectroscopy

**FIGURE 8:**
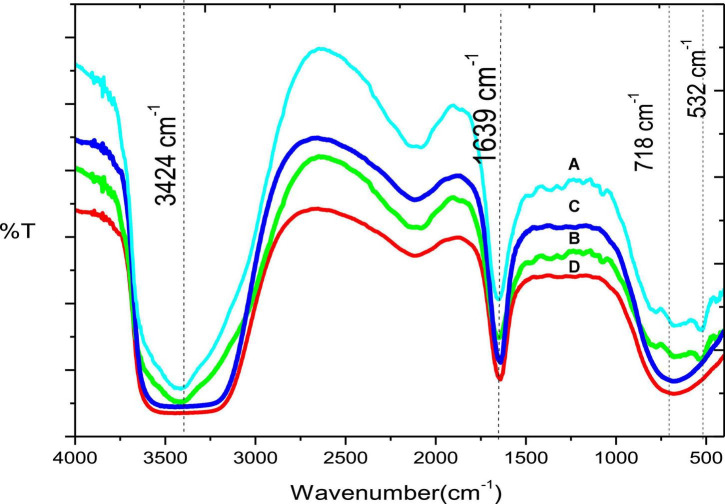
**(A)** IR Spectra of *Chromohalobacter* sp. grown at Mn amendments of 20 mM, **(B)** 2 mM, **(C)** without Mn, and **(D)** control of media only, without the microorganism or metal.

The IR spectra of *Chromohalobacter* sp. grown at Mn concentrations of (A) 20 mM, (B) 2 mM, (C) without Mn and (D) control of media only, without the microorganism or metal (Figure 8), indicated a peak at 3424 cm^–1^ due to presence of -OH stretching. This peak was present in all the samples, signifying the presence of -OH functionality. Absorption at 1639 cm^–1^ can be attributed to the presence of *C* = O functionality from amides which was present in all the samples. Absorption at 718 cm^–1^is due to the presence of Mn–oxygen stretching, and the one at 532 cm^–1^ is due to the presence of MnOx stretching seen in sample A and B; but absent in sample C and D.

### Screening for manganese oxidation genes

Preliminary evidence for the presence of genes for manganese oxidation in the genome of *Chromohalobacter* sp. was obtained by screening for *Mnx*G genes, which is the major gene involved in heavy metal tolerance to Mn. PCR with this degenerate primer yielded the expected ∼900 bp product (Supplementary Figure 4). The *Mnx* gene was obtained irrespective of the presence or absence of Mn during the growth of the cultures or in the reaction mixture suggesting that the Mn oxidizing activity could be constitutive.

### Bio-catalytic synthesis of n-butyl acetate

**FIGURE 9:**
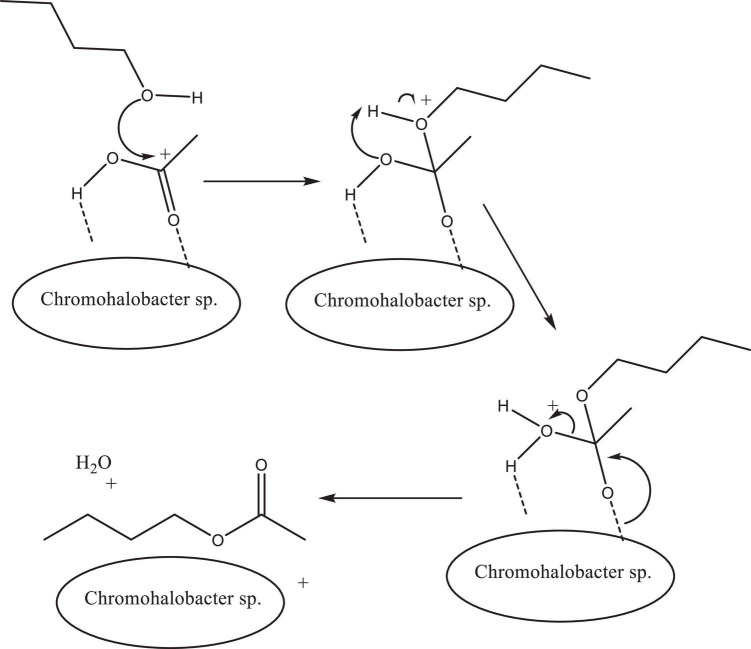
Plausible proposed mechanism of esterification of butan-1-ol and glacial acetic acid using *Chromohalobacter* sp. as catalyst.

Esterification of butan-1-ol with CH_3_COOH to produce n-butyl acetate using *Chromohalobacter* sp. showed a percentage conversion of ∼50% in 2 h at 70°C, which was found to be much higher than that shown by other chemical catalysts and metal oxides as depicted in Table 4. The probable mechanism for the reaction could be as proposed in Figure 9.

**TABLE 4:** Comparative table of various catalysts used for esterification reaction of butan-1-ol with CH_3_COOH.

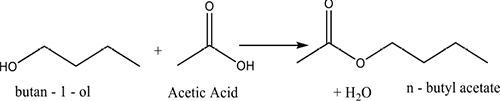					
Sr. no.	Catalyst	Catalyst weight	Reaction time (h)	% Conver-sion	References
1	ZnO	50 mg	2	42 ± 2	Nagvenkar et al., 2015
2	Zeolite	50 mg	2	47 ± 2	Nagvenkar et al., 2015
3	*Chromohalobacter* sp.	50 mg	2	50 ± 1.5	Present work

## Discussion

Environments continuously exposed to elevated concentrations of heavy metals are known to promote metal tolerance in bacteria (Clausen, 2000; Nosalova et al., 2023). The toxicity and solubility of metals is also known to be influenced by several physico-chemical parameters of that particular area as shown in Table 1, which plays an important role in developing metal tolerance in the indigenous microbial population (Shi et al., 2013; Gupta et al., 2023). This study was thus aimed at exploiting this hypothesis, for the isolation and screening of metal tolerant bacteria as potential isolates for bioremediation and other industrial applications. The State of Goa, in India is known to be a major exporter of Ferro-manganese ore, with a continuous influx of Fe and Mn into its estuaries during iron ore transport by barges. As shown in Figure 1A, the location of the Ribandar solar saltern is such that it is adjacent to and fed by the Mandovi estuary which faces the threat of metal contamination. Concentrations of Fe as high as 17.2 ± 2.8 to 26.3 ± 6.7 % and Mn at 0.6 ± 0.2 to 0.9 ± 0.2 % have been previously reported by Pereira et al. (2013) from the Ribandar saltern sediments. Metal-tolerance in bacteria is also influenced by metal speciation (Gadd, 1990; Li et al., 2022). In this study, the solubility of Mn in the saltern was found to be influenced by salinity, Eh and pH (Table 1). When the salinity of the water in the saltern increased, there was an increase in flocculation and coagulation of particulate material in suspension which in turn favored the quick settling of the particulates. This could be the reason why the Mn concentration obtained in this study was generally higher in the sediment than the overlying water. In the overlying water the pH varied from 7.0 to 8.5 and in the sediment pH varied between 5.7 and 7.4. A lower pH in the sediment could be responsible for the dissolution of the sedimented metals, thereby making them bioavailable to the surrounding microorganisms (Atkinson et al., 2007). Cyclic changes in pH and salinity in the overlying water are known to considerably influence metal liberation from estuarine sediments, thereby increasing the bioavailability of metals to the microbes, which subsequently results in a larger population of metal tolerant bacteria (Hong et al., 2011; Pereira et al., 2013; Swanson et al., 2023). Olaniran et al. (2013) demonstrated that even a small fluctuation in pH could influence the solubility and bioavailability of metals significantly, wherein they showed how the solubility of cadmium reduced by a factor>8 with an increase in pH from 6 to 7. In the present study, we observed that when the pH in the saltern increased from 7.4 to 8.4, it resulted in an approximately twofold decrease in the dissolved Mn. Largely positive values of Eh are known to favor the existence of oxidized metal species, whereas low values are known to favor the existence of reduced species. In the Ribandar saltern, the Eh values obtained were as low as −10, to as high as 122 mV. Positive values of Eh, coupled with low pH values may have resulted in higher levels of free ionic species of metals in the sediment. In the water, the detected concentrations of metal were lower and pH values were higher; resulting in negligible metal availability to the microbes. According to Grybos et al. (2022) the mobility of metals and its bioavailability to heterotrophic bacteria was greatly influenced by pH, Eh, temperature and the strain of microorganism under consideration. They reported a 40 times increase in Fe, Pb, and Mn concentration in the aquatic phase when pH decreased from 3.7 to 2.1. Thus this interplay of several factors could have influenced the heavy metal availability and the degree of metal toxicity in the saltern, resulting in the emergence of the multi-metal tolerant *Chromohalobacter* sp. Nosalova et al. (2023) in their study on mine tailings from a lead, silver and zinc mining heap noticed that the bacterial counts were low but showed exceptionally high levels of resistance to heavy metals. Our study showed a similar scenario as reported earlier (Pereira et al., 2013).

Growth rate kinetic analysis of bacteria was performed to understand the cellular processes that link two or more cellular functions, for example, the relationships between growth and cell doubling time, fitted using empirical functions by mathematical modeling based on multiple steps involved in the progression of cell growth (Tiwari et al., 2021). The experimental measurements requires the analysis of parameter statistics to estimate the precision of the results by applying descriptive methods. Virtually, all of the models developed for kinetic analysis are non-linear (Qu et al., 2004). These mathematical models describe the number of organisms or logarithm of the number of organisms as a function of time.

Growth curves obtained in UV-visible experiments can be fitted using various growth functions such as logistic and Gompertz model (Zwietering et al., 1990; Sprouffske and Wagner, 2016). The experimental optical density (O.D.) data is a function of time for control without any treatment. Bacterial growth often shows a phase in which the specific growth rate starts at a value of zero and then reaches a maximal value (μ_m_) in a certain period, resulting in a lag time (λ). The growth progress also contains a final phase in which the rate decreases and finally reaches zero, so that an asymptote (A) is reached or maximum O.D. As shown in Table 2, we fitted the growth curve of *Chromohalobacter* sp., treated with Manganese at varying salinities, employing the logistic and Gompertz function using Mathematica 9 software (Williams et al., 2012). These functions were found to be useful to obtain easily interpretable metrics such as population kinetics, the growth rate, the initial population size, and doubling time to summarize microbial growth curve data. In our study the changes in the growth rate showed sigmoidal behavior, with a lag phase immediately after *t* = 0, followed by an exponential phase and then a stationary phase.

The growth rate parameter (μ_m_), which controls the steepness of the function, is known to be the primary variable that describes the cellular growth. In our study, the growth rate parameter (μ_m_) showed a significant increase compared to the control, when salinity was enhanced to 10% (Supplementary Figure 2). With further increase in salinity to 20% there was a decrease in growth (Supplementary Figure 2).

Ventosa et al. (1998) reported that the requirement for salt and the limits of salt tolerance vary from species to species according to growth conditions, temperature and composition of media. In our study, *Chromohalobacter* sp. failed to grow in NaCl-deficient medium. Increase in the salinity to 2% (w/v) led to a greater tolerance to Mn. Further increase in salinity to 20 % enhanced tolerance to Mn from 0.01 to 5 mM. High salt tolerance of *Chromohalobacter* sp. could be due to the intrinsic adaptability of the organism to coastal saline soils (Ventosa et al., 1998). Similar findings were reported by Amoozegar et al. (2005). Osmotic stress was reported to trigger inhibition of ectoine transport at low salinity in *C. salexigens* Tn1732-induced mutant (CHR95) resulting in slower growth of the organism (Rodríguez-Moya et al., 2010). Enhanced growth of our *Chromohalobacter* sp. in the presence of manganese, as well as its potential to show multi-metal tolerance at high salinity could well be an indicator that our isolate had adapted to extremes of salinity and metal stress.

The Mn tolerant isolate FSK3 was identified as *Chromohalobacter* sp by 16 S rRNA sequencing (Table 3) *Chromohalobacter* spp. are known to be halophilic and capable of growing optimally at salinities of 8–10% NaCl, Our *Chromohalobacter* sp. was a borderline halophile capable of growing at salt concentrations up to 20%. It could grow over a temperature range of 25–55°C and pH 5.5–8.5, respectively. The preferred carbohydrate substrates utilized by *Chromohalobacter* sp, in the absence of Mn were Fructose, Gentobiose and Glucose, whereas the substrate utilization pattern changed with Mn amendments to D-Melibiose, Gentobiose and N Acetyl D-Glucosamine. It could not utilize lactose, xylose, maltose and mannitol. It was oxidase, catalase and urease positive. The organism produced a brown precipitate on Mn amended media, characteristic of it being a Manganese oxidizer. The first report of salt loving *Chromohalobacter* sp, was by T. Hof. It was isolated from fermented salted beans preserved in brine (Peçonek et al., 2006). The present study is the first report of a halophilic metal tolerant *Chromohalobacter* species from a solar saltern.

In the current study, metal tolerance in *Chromohalobacter* sp. could be due to an interplay of diverse mechanisms such as the secretion of EPS (Figure 4B), reduction in cell size (Figure 5B) and expression of *Mnx* genes (Supplementary Figure 4), resulting in the precipitation of Mn as MnO (Figure 6B). Bacterial polysaccharides have been reported by other authors to rapidly adsorb a range of metal ions such as Cd(II), Cu(II), Ni(II), and Zn(II) (Geddie and Sutherland, 1993). The anionic carboxyl and hydroxyl groups of EPS, a secondary metabolite, have powerful cation chelating properties. Further, the present *Chromohalobacter* sp. showed the presence of *Mnx*G genes in the chromosome implying a complexity of genes involving vertical inheritance and horizontal gene transfer. Haritha et al. (2009) suggested a strong possibility of acute metal stress triggering transposition of metal tolerance genes from plasmid to chromosome, thereby disseminating metal resistance traits. Chromosomal resistance therefore is expected due to continuous metal exposure. Dick et al. (2008) tentatively identified the putative enzymes multi-copper oxidases (MCOs) encoded by the *Mnx* gene to trigger Mn(II) oxidation in *Bacillus*, *Pseudomonas*, and *Leptothrix* spp., suggesting a universal mechanism of bacterial Mn(II) oxidation. MCOs are a family of enzymes that utilize four Copper ion cofactors to catalytically oxidize a wide range of substrates such as metals and organic compounds (Sakurai and Kataoka, 2007). In the present study, the oxide produced by *Chromohalobacter* sp. could be of Mn(IV) oxide, since it is more likely to be deposited on the bacterial surface on prolonged incubation (Ehrlich, 1999). Similar to this study, Francis and Tebo (2002) had reported oxidation of manganese by a spore forming marine *Bacillus* sp. strain SG-1 where Mn(II) oxidation was catalyzed by a multi-copper oxidase, MnxG. Zhang et al. (2014) demonstrated the role of Fe and Mn oxides in the sorption of other toxic elements like arsenic and elucidated that they could be highly efficient, economical, and environment friendly oxidants of toxic metals capable of environmental remediation. Our *Chromohalobacter* sp. showed ability to remove metals, well above the range of metal concentrations encountered in the saltern water (Figure 3A). Maximum bioremoval of Mn (76%) by *Chromohalobacter* sp. was at 0.1 mM at a rate of 8 mg Mn/g/day. Similar bioremediation of water contaminated with metals such as Fe, Mn, Cu, As, and Zn, by species from the *Ralstonia* family, i.e., *Ralstonia eutropha*, phylogenetically related to *Cupriavidus* was reported by Mondal et al. (2008), with removals of up to 65.2, 72.7, 98.6, 8, and 99.3%,respectively. Mn(III, IV) oxides and Mn complexes which are soluble are known to be the strongest oxidizing agents in the environment followed by oxygen and thereby play a key role in the biogeochemical cycles of major (C and S) and trace elements (Fe, Co, Pb, Cu, Cd, and Cr) (Laha and Luthy, 1990; Dick et al., 2008). Literature reveals biogeochemical cycling of Mn oxides to control the distribution of many trace elements, resulting in the adsorption and concentration of diverse metals. This is due to the highly charged nature of Mn minerals (Huang, 1991). In aerobic natural waters (pH 6 to 8), chemical oxidation of Mn (II) is slow, but in the presence of Mn (II)-oxidizing microorganisms, the rate is 4 to 5 times higher (Nealson et al., 1988; Wehrli et al., 1995; Stewart et al., 2007). Consequently, Mn oxidation could provide several pathways for transformation/sequestration of metal and organic contaminants. Hence, it is relevant that *Chromohalobacter* sp. capable of precipitating manganese as oxides, could also be capable of adsorbing or precipitating metals other than Mn, thus regulating and detoxifying the concentration of other metals as well in the salterns. *Chromohalobacter* sp. therefore could find applicability as an ideal model organism for manganese removal from ferro-manganese contaminated sites in estuaries and hypersaline environments. Wani et al. (2023) conducted *in vitro* studies and documented the use of heterotrophic bacteria as bio-inoculants beneficial in the removal of metal pollutants.

Further studies on mechanism of metal tolerance using AFM with Mn and crude salt also indicated binding of metal to the bacterial surface, as evidenced by the outer membrane roughness in the height profile (amplitude imaging) compared to the control (Figure 7). The exact mechanism of how these particles interact with the cell surface either directly or indirectly still remains unexplored. This study needs to be extended further to identify the exact concentration and other parameters.

The IR data confirmed presence of Mn (Figure 8) *Chromohalobacter* sp. grown at Mn amendments of 20 mM (sample A), and 2 mM (sample B), signifying that there is metal present in these samples which is absent in the other two samples. The existence of the peak at 718 cm^–1^ indicated the presence of metal–oxygen stretching in tetrahedral environment. This peak was absent in samples C and D, confirming that these samples did not have metal oxide. Presence of hydroxyl, amidic and carbonyl functionalities assist in the metal binding and in the formation of metal oxides. Swanson et al. (2023) demonstrated that alteration in the oxidation states of Fe and Mn, as would be the case in the Ribandar Saltern due to fluctuations in pH, temperature and organic matter, would cause temporary release of Fe and Mn into the surrounding, resulting in a larger population of metal tolerant bacteria capable of immobilizing Mn as manganese oxide. Thus, it can be seen that our *Chromohalobacter* sp. being capable of extracellular deposition of Mn, biosorption by EPS as well as oxidation of Mn could well be playing a crucial role in the remediation of metal pollutants in the saltern.

Another noteworthy application of this *Chromohalobacter* sp. is as a biocatalyst in the synthesis of n-butyl acetate from n-butyl alcohol. It is significant considering that this hypersaline, metal tolerant and high temperature tolerant organism could act as a biocatalyst capable of 50% conversion which is similar to that reported using chemical catalysts, but the noteworthy thing is that it could do so at a temperature less than 70°C, where Chen et al. (2011) reported the requirement of 1.5 g chemical catalyst and a temperature of 135°C for 4.5 h for the conversion, though the reported yield was higher. Our bio-catalyst was better than that reported by Nagvenkar et al. (2015) who used ZnO and Hβ zeolite as a catalyst but were able to obtain less than 50% conversion. Therefore, our *Chromohalobacter* sp. could be an excellent candidate for use as a bio-catalyst, since it exhibited catalytic activity for the synthesis of n-butyl acetate.

## Conclusion

Heavy metal accumulation in Ribandar salterns has affected the estuaries, as well as human health. Exposure to metals has resulted in emergence of metal tolerant bacteria. Multiple mechanisms have been suggested to get adjusted with fluctuations in metal concentrations. Presence of functional manganese tolerance genes could be used as biomarkers for Mn contamination. Since the *Chromohalobacter* sp. was capable of tolerating and immobilizing high concentration of Mn in this hypersaline ecosystem, it could be a candidate organism for scavenging Mn ions from polluted euryhaline environments. Existence of such metal tolerant bacteria in the Ribandar salterns could thus provide a solution to the environmental problems. *Chromohalobacter* sp. showed a catalytic activity of ∼50% conversion for the synthesis of n-butyl acetate, which is comparable to the percentage conversions shown by other catalysts in the literature. Thus this halophilic *Chromohalobacter* species from estuarine coastal waters could serve as an excellent detoxifier of Manganese, as well as a novel bio-catalyst for synthesis of n-butyl acetate.

## Data availability statement

The datasets presented in this study can be found in online repositories. The names of the repository/repositories and accession number(s) can be found in the article/Supplementary material.

## Author contributions

FP: conceptualization, investigation, lab experiment, data analysis, writing—original draft, and revision of manuscript. SK: conceptualization, supervision, resources, and project administration. DD: lab experiment, data analysis, and writing—original draft. VG: AFM experiment and data analysis. All authors provided critical feedback, helped to shape the research, analyzed the manuscript, discussed the results, and contributed to the final version of the manuscript.
