# Supplementary material for: A halophilic Chromohalobacter species from estuarine coastal waters as a detoxifier of manganese, as well as a novel bio-catalyst for synthesis of n-butyl acetate
Source: Front Microbiol. 2023 Apr 12;14:1159018. doi: 10.3389/fmicb.2023.1159018 (PMC10130588; doi:10.3389/fmicb.2023.1159018)
Supplement: Supplementary file 1 [file Data_Sheet_1.pdf]

Supplementary Material

A halophilic *Chromohalobacter* species from estuarine coastal waters as a detoxifier of Manganese, as well as a novel bio-catalyst for synthesis of n-butyl acetate

Flory Pereira[1]*, Savita Kerkar[2] , Dominic Savio Dias[3]and Vivekanand V Gobre[4]

[1]Department of Microbiology, P. E. S's R. S. N. College of Arts & Science; Farmagudi, Ponda – Goa, India. 403401. florycliffy@gmail.com

[2]School of Biological Sciences and Biotechnology, Goa University, Taleigao Plateau, Goa, India. 403206. savita@unigoa.ac.in

[3]Department of Chemistry, P. E. S's R. S. N. College of Arts & Science; Farmagudi, Ponda – Goa, India. 403401 dominicsaviodias@gmail.com

[4]School of Chemical Sciences, Goa University, Taleigao Plateau, Goa, India.403206 vvgobre@unigoa.ac.in

1 Supplementary Figures and Tables

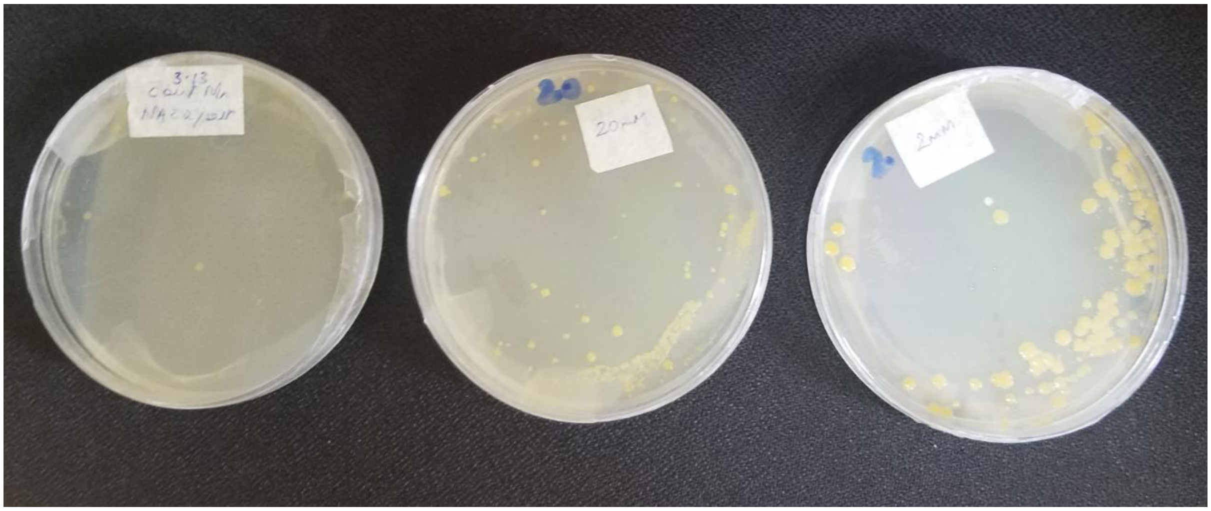

Figure S1 Growth and pigmentation of FSK3 without Mn and with 20 mM and 2mM Mn .

```
(*dataz
 t,0ppt,20ppt,100ppt,200ppt
0,0.055,0.045,0.045,0.060
1,0.099,0.096,0.094,0.099
2,0.107,0.155,0.342,0.211
3,0.121,0.270,0.471,0.355
4,0.140,0.291,0.448,0.367
5,0.181,0.324,0.441,0.385
6,0.200,0.344,0.418,0.380
7,0.221,0.341,0.408,0.370
8,0.231,0.326,0.399,0.363
9,0.240,0.317,0.384,0.356
10,0.270,0.312,0.361,0.347
11,0.263,0.307,0.356,0.341
*)

(*
Zwietering,M.H.,
et al."Modeling of the bacterial growth curve." Applied and environmental
  microbiology 56.6 (1990):1875-1881.
*)



datz = {{0, 0.055, 0.045, 0.045, 0.060}, {1, 0.099, 0.096, 0.094, 0.099},
   {2, 0.107, 0.155, 0.342, 0.211}, {3, 0.121, 0.270, 0.471, 0.355},
   {4, 0.140, 0.291, 0.448, 0.367}, {5, 0.181, 0.324, 0.441, 0.385},
   {6, 0.200, 0.344, 0.418, 0.380}, {7, 0.221, 0.341, 0.408, 0.370},
   {8, 0.231, 0.326, 0.399, 0.363}, {9, 0.240, 0.317, 0.384, 0.356}, {
10, 0.270, 0.312, 0.361, 0.347}, {11, 0.263, 0.307, 0.356, 0.341}};
```

$$\text{logistic} = \frac{A}{1 + \text{Exp}\left[\frac{4\,\mu_m}{A}\,(\lambda - t) + 2\right]};$$

$$\text{gompertz} = A\,\text{Exp}\left[-\text{Exp}\left[\frac{\mu_m\,\text{Exp}[1]}{A}\,(\lambda - t) + 1\right]\right];$$

$$\text{richards} = A\left(1 + \nu\,\text{Exp}[1 + \nu]\,\text{Exp}\left[\frac{\mu_m}{A}\,(1 + \nu)\left(1 + \frac{1}{\nu}\right)(\lambda - t)\right]\right)^{\frac{-1}{\nu}};$$

```
(*0 ppt*)
logfitc = FindFit[datz[[1 ;;, {1, 2}]], logistic,
  {{A, 0.19}, {μm, 0.98}, {λ, 0.89}}, t]
gomfitc = FindFit[datz[[1 ;;, {1, 2}]], gompertz,
  {{A, 0.19}, {μm, 0.98}, {λ, 0.89}}, t]

Show[ListPlot[datz[[1 ;;, {1, 2}]], PlotRange → All,
  PlotStyle → Directive[PointSize[0.03],
    PlotLegends → Placed[{"Group"}, {0.28, 0.9}], Black],
  AxesLabel → {Style["time (in days)", Black, FontFamily → "Zapfino",
     FontSize → 18], Style["Optical Density at 0% Salinity", Black,
     FontFamily → "Zapfino", FontSize → 18]}],
 Show[Plot[Evaluate[logistic /. logfitc], {t, 0, 10},
   PlotLegends → Placed["Expressions", {0.28, 0.9}],
   PlotStyle → {Thick, Dashed, Red}]],
 Show[Plot[Evaluate[gompertz /. gomfitc], {t, 0, 10},
   PlotLegends → Placed["Expressions", {0.28, 0.8}],
   PlotStyle → {Thick, Dotted, Blue}]], ImageSize → 800]
```

$\{A \to 0.292836,\ \mu_m \to 0.0240629,\ \lambda \to -2.27878\}$

$\{A \to 0.328095, \ \mu_m \to 0.0236639, \ \lambda \to -2.48301\}$

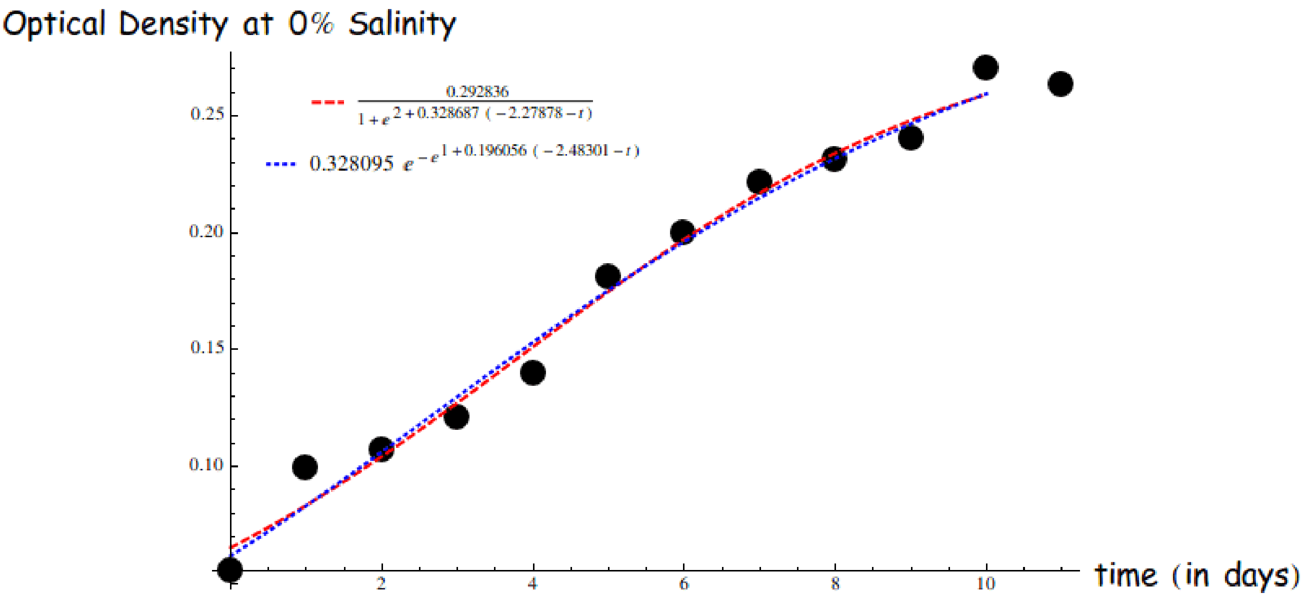

(*20 ppt*)

```
logfita = FindFit[datz[[1 ;;, {1, 3}]], logistic, {A, μm, λ}, t]
gomfita = FindFit[datz[[1 ;;, {1, 3}]], gompertz, {A, μm, λ}, t]
Show[ListPlot[datz[[1 ;;, {1, 3}]], PlotRange → All,
  PlotStyle → Directive[PointSize[0.03],
    PlotLegends → Placed[{"Group"}, {0.28, 0.9}], Black],
  AxesLabel → {Style["time (in days)", Black, FontFamily → "Zapfino",
     FontSize → 18], Style["Optical Density at 2% Salinity", Black,
     FontFamily → "Zapfino", FontSize → 18]}],
 Show[Plot[Evaluate[logistic /. logfita], {t, 0, 10},
   PlotLegends → Placed["Expressions", {0.28, 0.9}],
   PlotStyle → {Thick, Dashed, Red}]],
 Show[Plot[Evaluate[gompertz /. gomfita], {t, 0, 10},
   PlotLegends → Placed["Expressions", {0.28, 0.8}],
   PlotStyle → {Thick, Dotted, Blue}]], ImageSize → 800]
```

$\{A \to 0.32608, \ \mu_m \to 0.0913712, \ \lambda \to 0.0887089\}$

$\{A \to 0.328924, \ \mu_m \to 0.0933111, \ \lambda \to -0.013241\}$

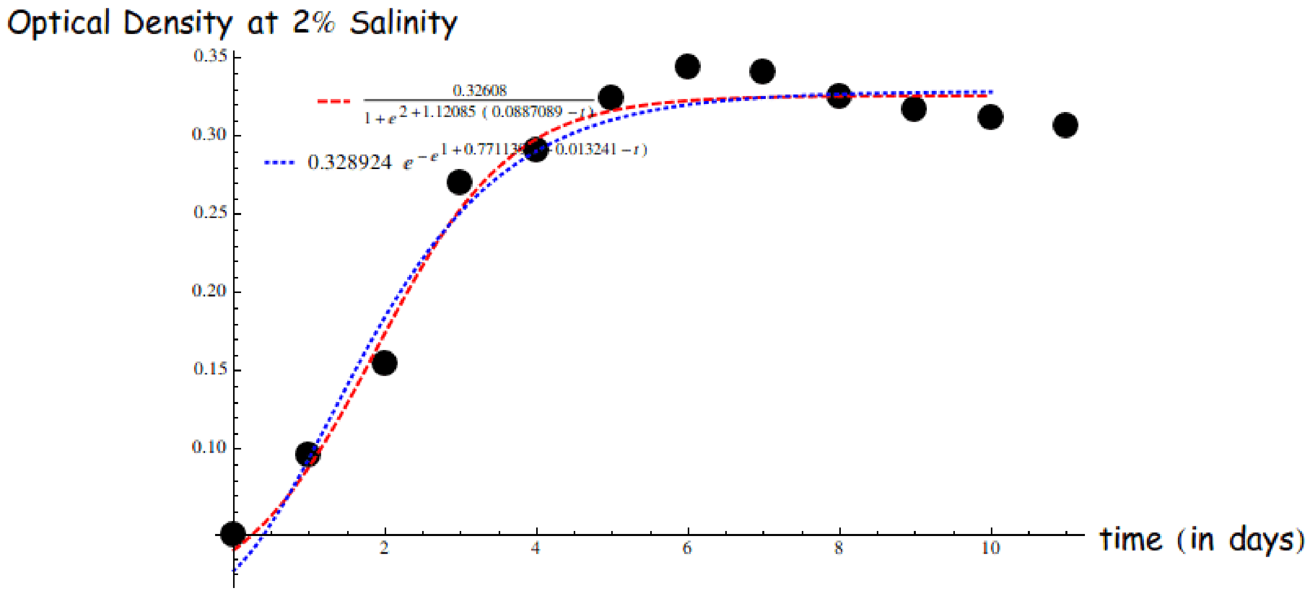


```
(*100 ppt*)
logfitb = FindFit[datz[[1 ;;, {1, 4}]], logistic,
  {{A, 0.19}, {μm, 0.98}, {λ, 0.89}}, t]
gomfitb = FindFit[datz[[1 ;;, {1, 4}]], gompertz,
  {{A, 0.19}, {μm, 0.98}, {λ, 0.89}}, t]
Show[ListPlot[datz[[1 ;;, {1, 4}]], PlotRange → All,
  PlotStyle → Directive[PointSize[0.03],
    PlotLegends → Placed[{"Group"}, {0.28, 0.9}], Black],
  AxesLabel → {Style["time (in days)", Black, FontFamily → "Zapfino",
     FontSize → 18], Style["Optical Density at 10% Salinity", Black,
     FontFamily → "Zapfino", FontSize → 18]}],
 Show[Plot[Evaluate[logistic /. logfitb], {t, 0, 10},
   PlotLegends → Placed["Expressions", {0.28, 0.9}],
   PlotStyle → {Thick, Dashed, Red}]],
 Show[Plot[Evaluate[gompertz /. gomfitb], {t, 0, 10},
   PlotLegends → Placed["Expressions", {0.28, 0.8}],
   PlotStyle → {Thick, Dotted, Blue}]], ImageSize → 800]
```

$\{A \to 0.409422,\ \mu_m \to 0.295899,\ \lambda \to 0.711574\}$

$\{A \to 0.408681,\ \mu_m \to 0.361577,\ \lambda \to 0.7525\}$

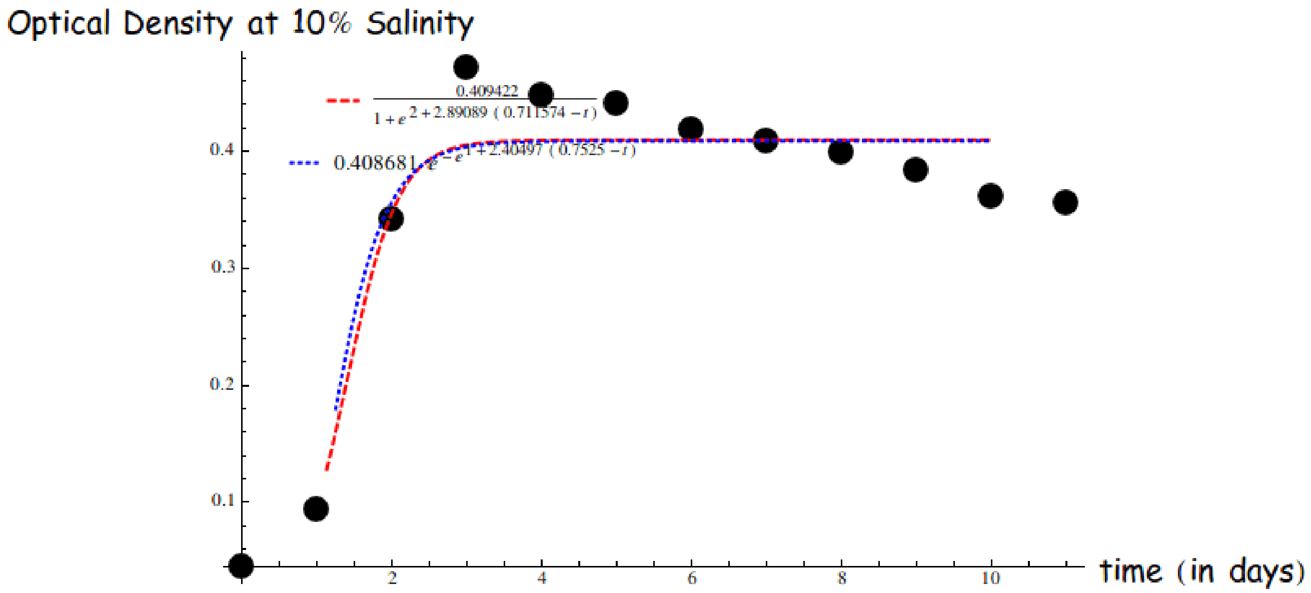


```
(*200 ppt *)
logfitd = FindFit[datz[[1 ;;, {1, 5}]], logistic, {A, μm, λ}, t]
gomfitd = FindFit[datz[[1 ;;, {1, 5}]], gompertz, {A, μm, λ}, t]
Show[ListPlot[datz[[1 ;;, {1, 5}]],
  PlotStyle → Directive[PointSize[0.03],
    PlotLegends → Placed[{"Group"}, {0.28, 0.9}], Black],
  AxesLabel → {Style["time (in days)", Black, FontFamily → "Zapfino",
     FontSize → 18], Style["Optical Density at 20% Salinity", Black,
     FontFamily → "Zapfino", FontSize → 18]}],
 Show[Plot[Evaluate[logistic /. logfitd], {t, 0, 10},
   PlotLegends → Placed["Expressions", {0.28, 0.9}],
   PlotStyle → {Thick, Dashed, Red}]],
 Show[Plot[Evaluate[gompertz /. gomfitd], {t, 0, 10},
   PlotLegends → Placed["Expressions", {0.28, 0.8}],
   PlotStyle → {Thick, Dotted, Blue}]], ImageSize → 800]
```

$\{A \to 0.366943,\ \mu_m \to 0.13841,\ \lambda \to 0.308756\}$

$\{A \to 0.368122,\ \mu_m \to 0.148003,\ \lambda \to 0.300333\}$

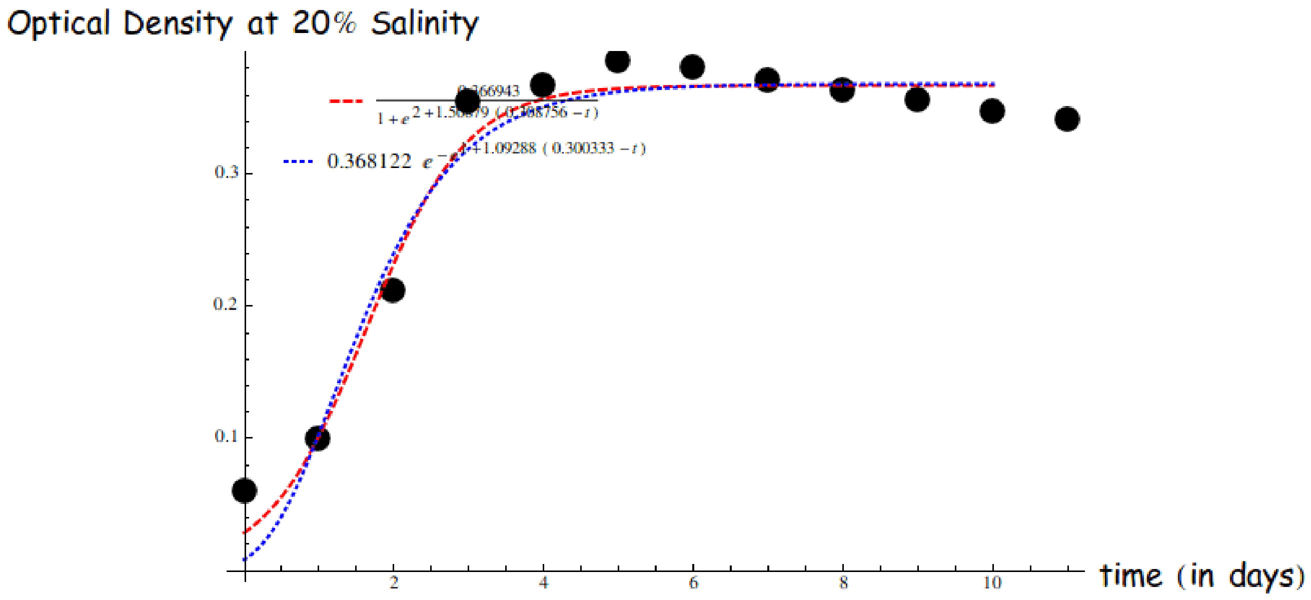

Figure S2 Optical density of *Chromohalobacter* sp. at varying salinities versus time in days modelled using Mathematica 9 software .

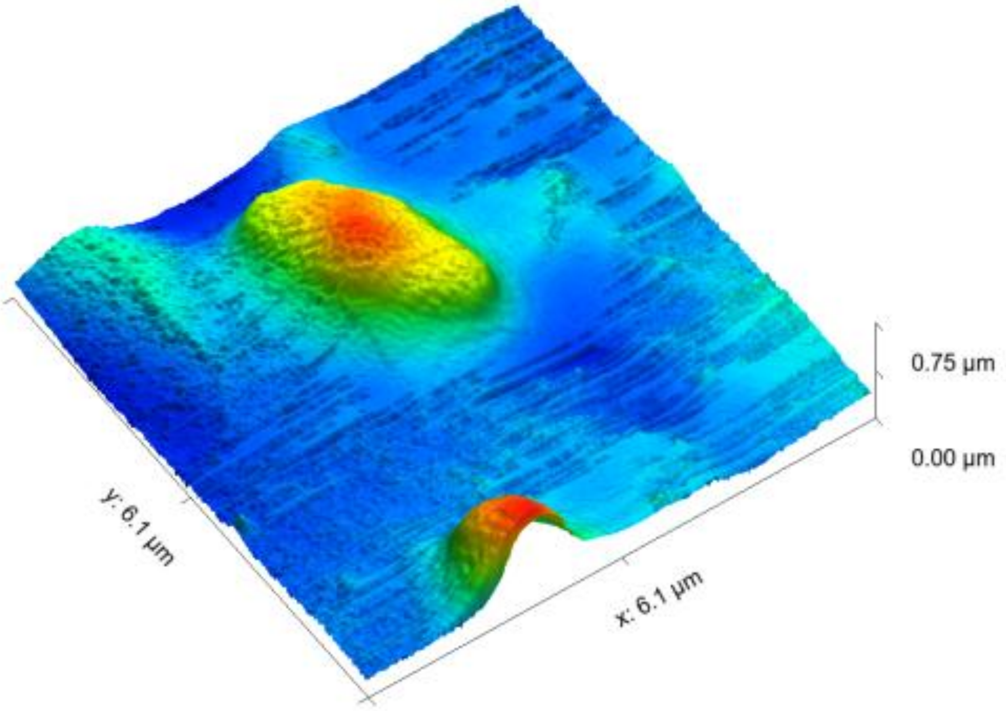

0.75 µm
0.00 µm
y: 6.1 µm
x: 6.1 µm

A

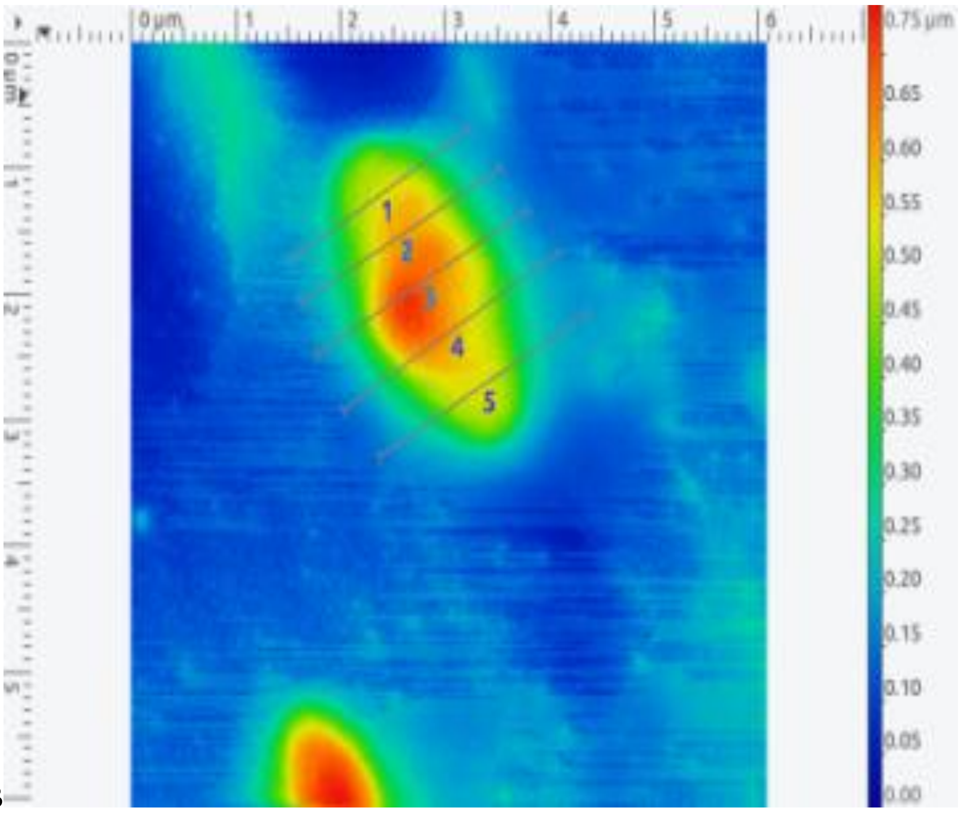

0 µm
1
2
3
4
5
6
0.75 µm
0.65
0.60
0.55
0.50
0.45
0.40
0.35
0.30
0.25
0.20
0.15
0.10
0.05
0.00

B

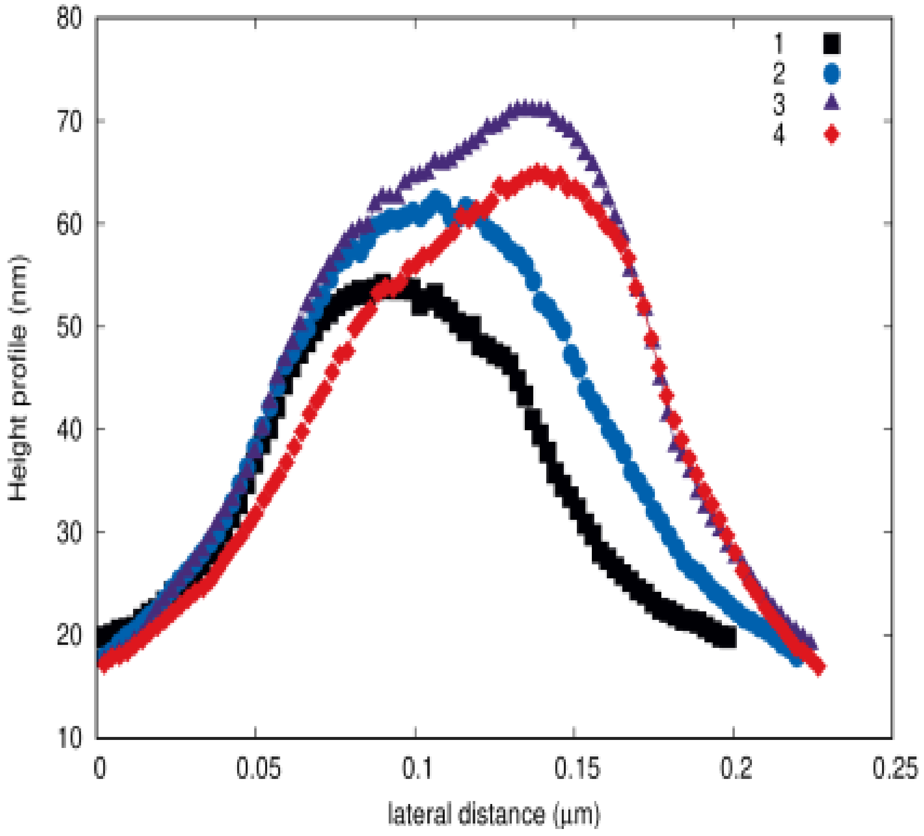

Height profile (nm)
lateral distance (μm)
1
2
3
4

C

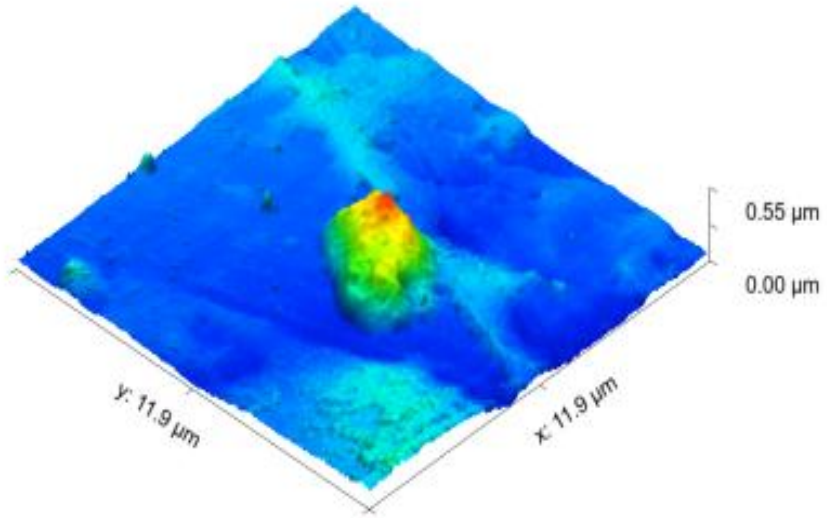

0.55 μm
0.00 μm
y: 11.9 μm
x: 11.9 μm

D

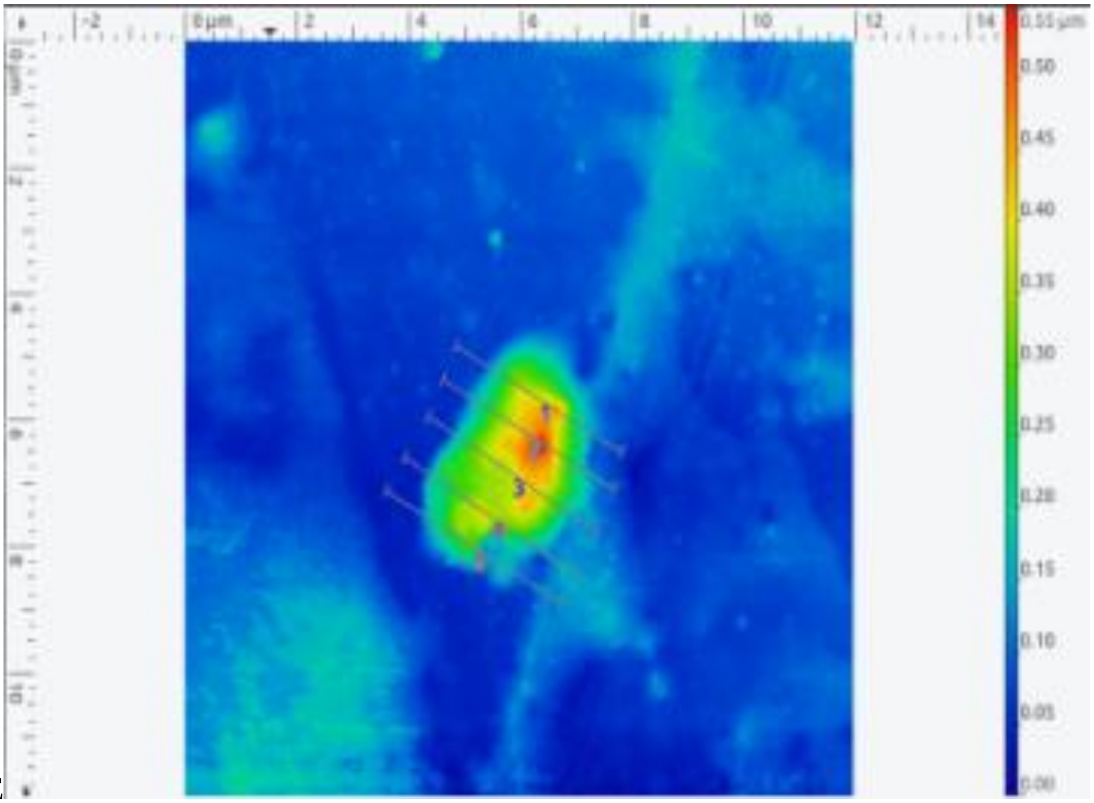

E

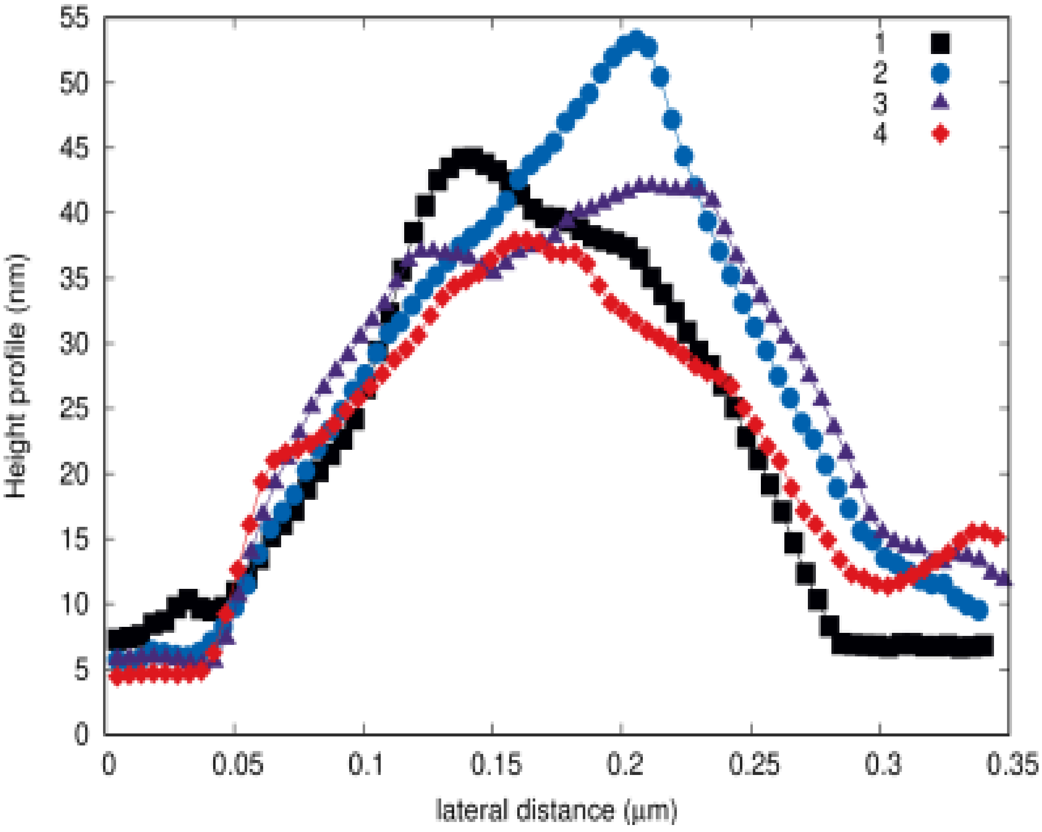

Height profile (nm)
lateral distance (µm)
1
2
3
4
0
5
10
15
20
25
30
35
40
45
50
55
0
0.05
0.1
0.15
0.2
0.25
0.3
0.35

F

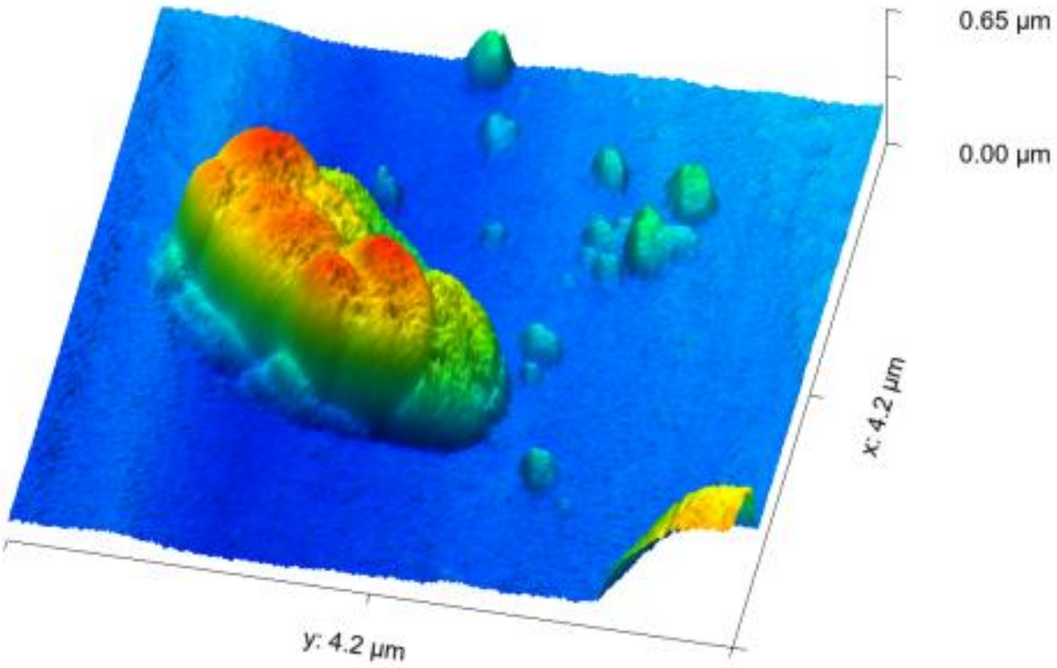
0.65 µm
0.00 µm
x: 4.2 µm
y: 4.2 µm

G

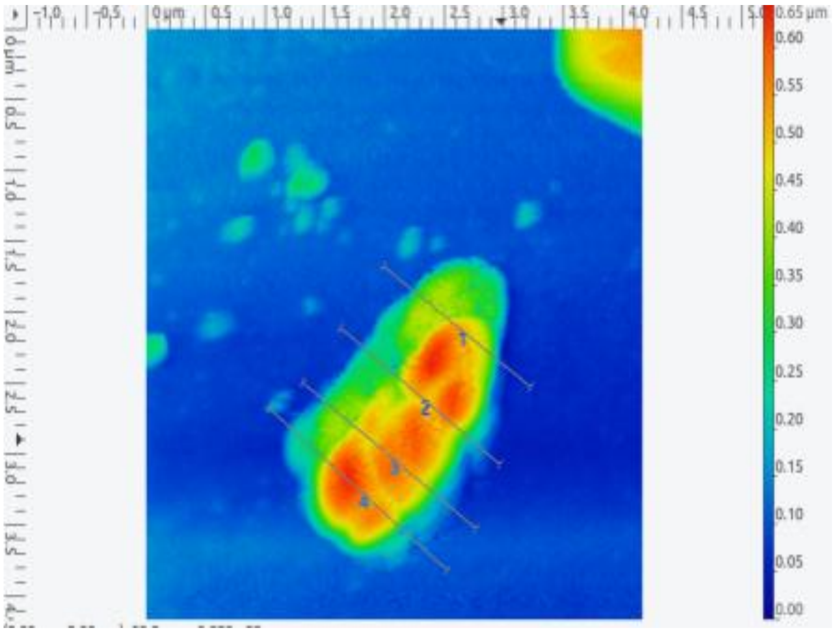
-1.0
-0.5
0 µm
0.5
1.0
1.5
2.0
2.5
3.0
3.5
4.0
4.5
5.0
0.65 µm
0.60
0.55
0.50
0.45
0.40
0.35
0.30
0.25
0.20
0.15
0.10
0.05
0.00
1
2
3
4

H

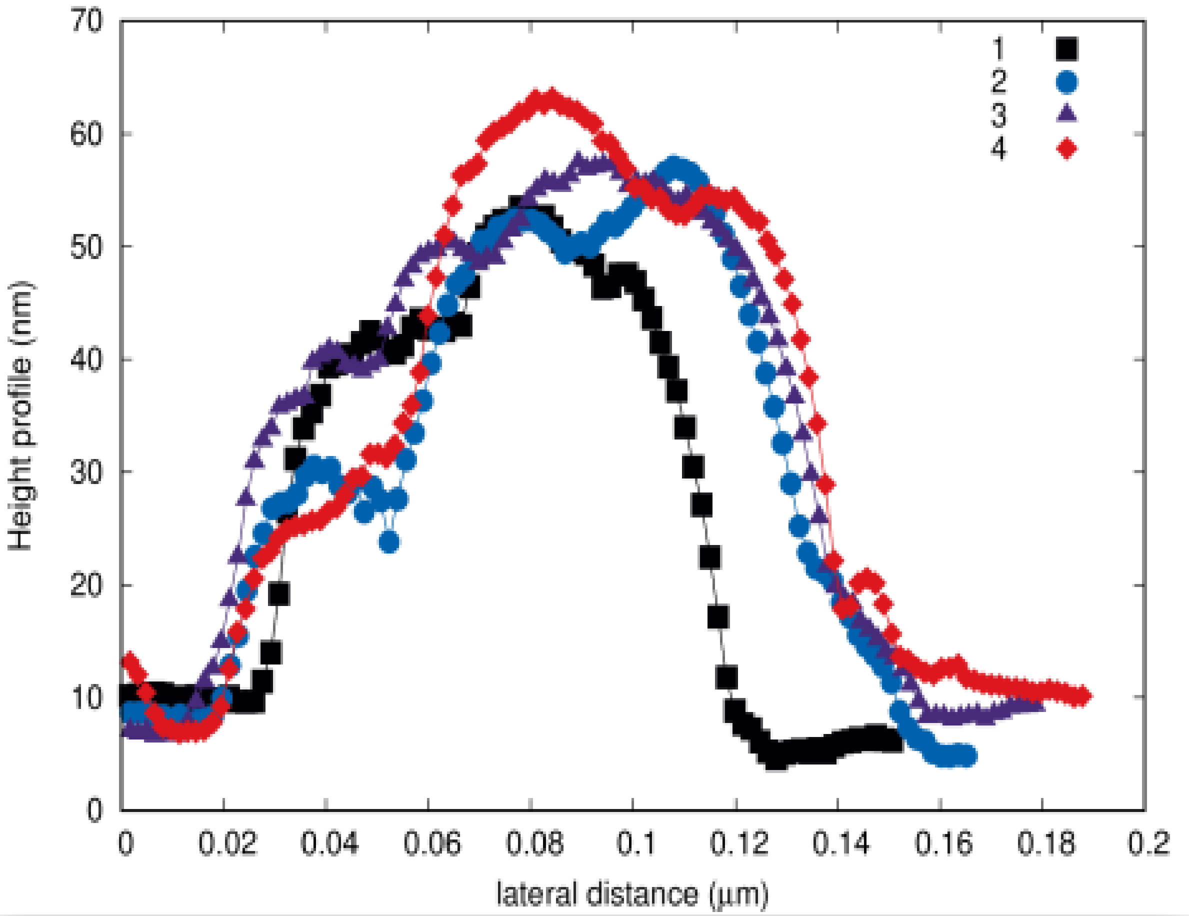

I

Figure S3 *Chromohalobacter* single cell surface topography as mapped under AFM. (A–C) Control without any treatment. *Chromohalobacter* cells treated with 20mM concentration of Mn (D-F). *Chromohalobacter* cells treated with 2mM concentration of Mn (G-I), where (A-D-G) indicate NC-AFM 3D Height profile, (B-E-H) linear path mapping across single cell, (C-F-I) height profile graph of surface roughness for *Chromohalobacter* samples.

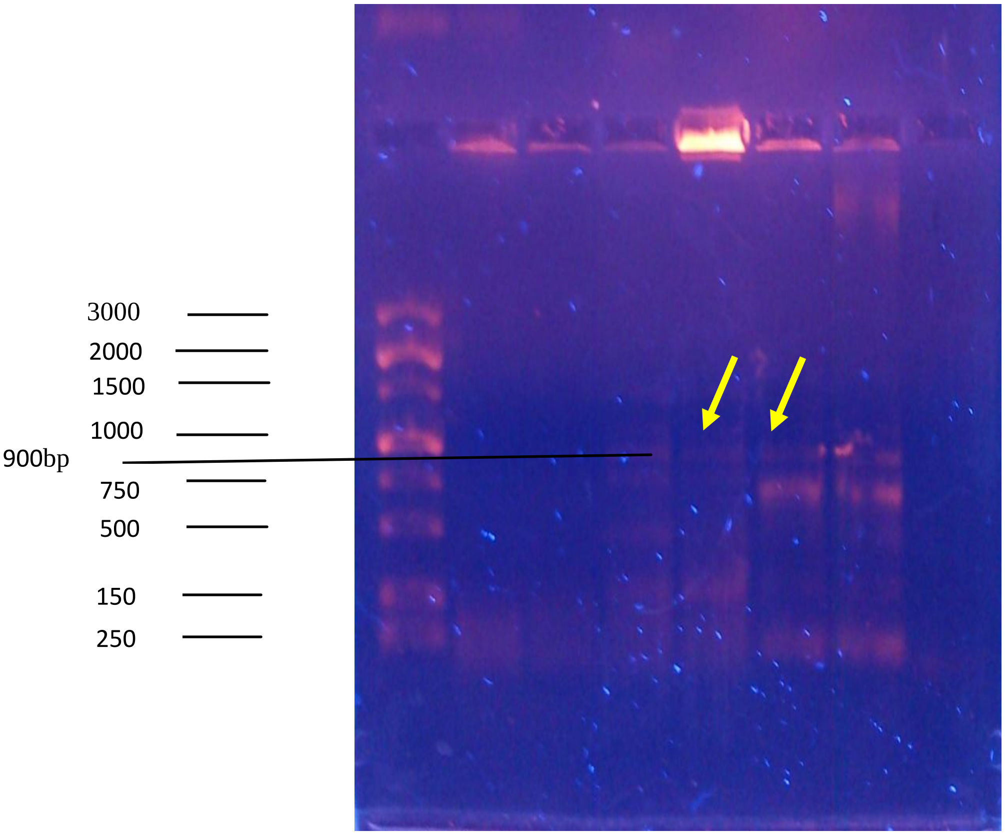

Figure S4 Agarose gel electrophoresis of mnxG PCR products with and without Mn amendments. Lane 1 - Marker, lanes 2,3,4,5 - other isolates, lane 6 - *Chromohalobacter* sp. without Mn, lane 7 - *Chromohalobacter* sp. with Mn.
